# A medication-wide association study (MWAS) on repurposed drugs for COVID-19 with Pre-pandemic prescription medication exposure and pregnancy outcomes

**DOI:** 10.1038/s41598-022-24218-1

**Published:** 2022-11-24

**Authors:** Lena Davidson, Silvia P. Canelón, Mary Regina Boland

**Affiliations:** 1 Department of Biostatistics, Epidemiology and Informatics, Perelman School of Medicine, University of Pennsylvania, 423 Guardian Drive, 421 Blockley Hall, Philadelphia, PA 19104 USA; 2 Institute for Biomedical Informatics, University of Pennsylvania, Philadelphia, USA; 3 Center for Excellence in Environmental Toxicology, University of Pennsylvania, Philadelphia, USA; 4 Department of Biomedical and Health Informatics, Children’s Hospital of Philadelphia, Philadelphia, USA

**Keywords:** Public health, Drug therapy, Health policy

## Abstract

Information on effects of medication therapies during pregnancy is lacking as pregnant patients are often excluded from clinical trials. This retrospective study explores the potential of using electronic health record (EHR) data to inform safety profiles of repurposed COVID medication therapies on pregnancy outcomes using pre-COVID data. We conducted a medication-wide association study (MWAS) on prescription medication exposures during pregnancy and the risk of cesarean section, preterm birth, and stillbirth, using EHR data between 2010–2017 on deliveries at PennMedicine. Repurposed drugs studied for treatment of COVID-19 were extracted from ClinicalTrials.gov (n = 138). We adjusted for known comorbidities diagnosed within 2 years prior to birth. Using previously developed medication mapping and delivery-identification algorithms, we identified medication exposure in 2,830 of a total 63,334 deliveries; from 138 trials, we found 31 medications prescribed and included in our cohort. We found 21 (68%) of the 31 medications were not positively associated with increased risk of the outcomes examined. With caution, these medications warrant potential for inclusion of pregnant individuals in future studies, while drugs found to be associated with pregnancy outcomes require further investigation. MWAS facilitates hypothesis-driven evaluation of drug safety across all prescription medications, revealing potential drug candidates for further research.

## Introduction

At the break of the COVID-19 pandemic, little was known about the effects of this emerging disease on maternal or neonatal outcomes. Physiological changes that occur during pregnancy arise concerns for susceptibility and morbidity from contracting COVID-19, particularly respiratory distress. Multiple adverse pregnancy outcomes have been reported, including increased risk of respiratory failure, preterm birth, stillbirth, preeclampsia, and miscarriage^1–8^. Moreover, COVID-19 associated maternal deaths are likely undercounted nationally^9^.

Initially, research on treatments focused on previously approved antiviral agents, immunomodulating agents, and other therapeutic solutions to develop new treatments for COVID-19. In comparison to de novo drug discovery for COVID-19, drug repurposing provided a quick and lower cost way to identify new therapies. Drug repurposing is the reuse of an existing approved drug for a new clinical indication. Detailed information on the known safety profile (i.e., potential toxicity) provides another incentive to repurpose existing drugs. Repurposed drug therapies must be assessed carefully due to limited data available for the pregnant population, a population systematically excluded from pharmaceutical research^10^. Valid concerns regarding fetal and infant exposure have led to this knowledge gap. Reported prescription medication use in the pregnant population varies, with rates between 50–75%^11,12^. In practice, off-label medicines (i.e., medications used for indications outside of what the US Food & Drug Administration approved) are prescribed to pregnant patients^13^. Taylor et al. searched clinical trial registries during two time points from April to July 2020 and identified 75–80% of COVID-19 treatment studies explicitly excluded pregnant women^14^. In the COVID-19 pandemic, pregnant patients inevitably receive said therapies under investigation despite the lack of evidence.

Retrospective health record data has the potential to provide insight into pregnancy outcomes associated with the proposed repurposed drugs. Pregnant people and their unborn children can directly benefit from medication safety knowledge that arises from the analysis of retrospective electronic health record (EHR) data^15^. In efforts to close the gap between observational research and clinical trial research, epidemiologists have proposed methodology to emulate clinical trial research with retrospective data^16,17^. Others have proposed observing all medications in relation to an outcome of interest, proposing the medication-wide association study (MWAS)^18^. The MWAS approach is based upon genome- and phenome-wide association studies (GWAS and PWAS, respectively). Previous MWAS applications have observed medication side effects concerning cancer risk^19,20^; spontaneous preterm birth^21^; acute myocardial infarction^18,22,23^; acute liver failure, acute renal failure, and upper gastrointestinal ulcer^18^. A select few sourced data from nationwide healthcare data registries^19,20,22^, however the MWAS approach is often applied to administrative claims data^18,21,23^. In prior work, we explored the risk of multiple birth as a pregnancy outcome and demonstrated the potential of the MWAS approach with EHR data to generate hypotheses for future work^24^. In this work, each pregnancy outcome is compared with all drugs prescribed during pregnancy. We propose hypothesis-generating methodology to apply MWAS to retrospective EHR data to systematically examine associations between medications prescribed during pregnancy and the occurrence of pregnancy outcomes (specifically, cesarean section, preterm birth, and stillbirth) of patients who have delivered at Penn Medicine. The resulting medication-outcome information has the potential to inform further study for  the inclusion and exclusion of pregnant people in clinical research trials of repurposed drugs.

## Methods

### Data source & identification of stillbirth, preterm birth & cesarean section (outcome)

We used EHR data obtained from 4 different hospitals within the Penn Medicine system, the Hospital of the University of Pennsylvania (HUP), the Pennsylvania Hospital, Penn Presbyterian Hospital, and Chester County Hospital. These deliveries occurred between 2010 and 2017. Deliveries were identified using a previously developed algorithm called MADDIE: Method to Acquire Delivery Date Information from Electronic Health Records^25^. Pregnancy outcomes are the result of conception and ensuing pregnancy (e.g. live birth, spontaneous abortion, birth weight). For the purposes of this study, the pregnancy outcomes studied are cesarean section, preterm birth, and stillbirth, as determined by The International Classification of Diseases, 9th Revision, Clinical Modification (ICD-9-CM) and ICD, 10th Revision (ICD-10) billing codes (See Supplemental Table S1). Numerous codes are related to stillbirth, preterm birth, and cesarean section. However, some are not explicit that a delivery occurred (i.e. ICD-9 code 651.03) and therefore we did not include these codes.

#### Regulatory information

All methods described in this paper were carried out in accordance with relevant guidelines and regulations. The institutional review board (IRB) at Penn Medicine approved this study, which is a retrospective analysis of existing clinical records. Because this study is being conducted as a retrospective analysis of existing clinical records, a waiver of consent was obtained from the IRB at Penn Medicine. All patient records were stored and maintained on HIPAA secure servers and managed by institutional Information Technology (IT) teams to maintain compliance with regulatory protocols.

### Drug classification (exposure classification)

#### Repurposed drug classification

We queried ClinicalTrials.gov on 31 July 2020 for drugs repurposed for COVID-19 treatment, filtered by study type interventional. The search included the following terms: COVID-19, COVID, SARS-CoV-2, severe acute respiratory syndrome coronavirus 2, 2019-nCoV, 2019 novel coronavirus, and Wuhan coronavirus. With the resulting CSV file, we manually labeled medications used in each trial. We excluded trials researching experimental drugs without United States Food & Drug Administration (US FDA) approval, drugs approved after 2017, and non-pharmaceutical or biological products (e.g., convalescent plasma). Trials of antiretroviral medications with the sole US FDA approved indication for treatment of Human Immunodeficiency Virus (e.g. lopinavir) were excluded as HIV status and proxy to such status is not included in this study. Moreover, we excluded trials without female participants of reproductive age.

#### Pregnancy exposure drug classification

We mapped all inpatient and outpatient medications, sourced from EPIC and other EHR systems, to RxNorm using a method described previously^26^. We defined a ‘pregnancy exposure’ as one or more medication prescriptions occurring from 280 days before delivery up to 1 day before delivery. In order to ensure patient privacy and avoid the low statistical power of uncommonly prescribed medication-outcome associations, we only included medications prescribed to ten or more patients within the pregnancy exposure time.

We manually annotate the following for the complete medication list: generic name, medication type, specific medication type, and associated comorbidities. We made reference of Drugs.com Database^27^, RxNav^28^, and a guide to fetal and neonatal risk^29^ to assign these qualities to each medication as appropriate. The Drugs.com Drug Information Database is sourced from several medication information suppliers, including Wolters Kluwer Health, American Society of Health-System Pharmacists, Cerner Multum, IBM Watson Micromedex, and Mayo Clinic^27^.

### Statistical analysis: medication-wide association study of pregnancy outcomes

We built three logistic regression models, one for each outcome of interest (binary outcome): cesarean section, stillbirth, and preterm birth. Each medication’s effect on the outcome was assessed separately (each medication exposure was a binary variable). Medication exposures to generic and brand name equivalent drugs were also merged (e.g., oseltamivir & Tamiflu). We report odds ratios (OR) with 95% confidence intervals and significance where the nominal *P*-value is less than 0.05. Additionally, we perform the conservative Bonferroni correction to decrease the risk of type 1 error (i.e. false positive). Indication of medication prescription is outside the scope of this analysis. Therefore, we adjusted maternal age and for 16 covariates known to be associated with increased maternal and fetal complications at the time of delivery. These 16 covariates are infectious disease, obesity, cancer, cardiovascular disease, circulatory disease, cerebrovascular disease, respiratory disease, immune disorders, organ transplant, obstetric history, maternal care, preeclampsia, multiple birth, drug allergies, procedures, and drug resistance. These covariates were associated with high-risk pregnancies and delivery outcomes from the literature (See Supplemental Table S2). We used ICD-9 and ICD-10 codes to identify patients as having these covariates from the EHR (See Supplemental Table S3). Medications prescribed during the exposure window may be due to a diagnosis assigned outside of our observed exposure window. To overcome such missingness in the EHR data, we required the diagnosis to have occurred within 2 years before the delivery. We did not include diagnoses made on the day of delivery as these are often related to the outcome and delivery itself and therefore are not ‘pre-existing conditions’.

## Results

### Cohort characteristics

We obtained EHR data from 1,060,100 female patients treated at Penn Medicine, with inpatient and outpatient visits between 2010–2017. A previously developed algorithm called MADDIE: Method to Acquire Delivery Date Information from Electronic Health Records identified 50,560 patients having 63,334 distinct deliveries^25^. Our cohort contains 63,334 pregnancies delivered between 2010–2017 at Penn Medicine, which includes 4 distinct hospitals located in Philadelphia (3 hospitals) and Chester County (Greater Philadelphia Area)^25^. Shown in Table 1, of 63,334 deliveries there were 3,897 with preterm birth (6.15%), 516 pregnancies resulted in stillbirth (0.81%), and 20,894 pregnancies resulted in a cesarean Sect. (32.99%). We found that 12,251 deliveries had a recorded prescription medication exposure during pregnancy (19.34%), of which 2,830 pregnancies (4.47%) were exposed to medications that were extracted from our clinical trial query. Of those with prenatal medication exposure (N = 2,830), 29 (1.02%) resulted in stillbirth, 296 (10.46%) resulted in preterm birth, and 1,038 (36.68%) resulted in cesarean section. Shown in Table 1, the most common comorbidity diagnoses were preeclampsia (n = 5071, 8.01%), multiple birth (n = 1562, 2.47%), and infectious disease (n = 1255, 1.98%), respectively.

**Table 1:** Cohort of deliveries with and without medication exposure to repurposed medications.

	No medication exposure (N = 60,504)		Medication exposure (N = 2,830)		Total distinct deliveries (N = 63,334)	
	N	%	N	%	N	%
**Pregnancy outcome**						
Cesarean section	19,856	32.82	1038	36.68	20,894	32.99
Preterm birth	3601	5.95	296	10.46	3897	6.15
Stillbirth	487	0.80	29	1.02	516	0.81
**Comorbidities for adjustment**						
Adverse drug	27	0.04	*	0.32	36	0.06
Autoimmune	27	0.04	18	0.64	45	0.07
Cancer	312	0.52	85	3.00	397	0.63
Cardiovascular	411	0.68	202	7.14	613	0.97
Cerebrovascular	*	<0.01	*	0.11	*	0.01
Circulatory	18	0.03	29	1.02	47	0.07
Infectious disease	965	1.59	290	10.25	1255	1.98
Maternal care	214	0.35	50	1.77	264	0.42
Medication allergy	231	0.38	79	2.79	310	0.49
Multiple birth	1415	2.34	147	5.19	1562	2.47
Obesity	892	1.47	209	7.39	1101	1.74
Obstetric history	121	0.20	61	2.16	182	0.29
Organ transplant/acquired absence	20	0.03	18	0.64	38	0.06
Preeclampsia	4718	7.80	353	12.47	5071	8.01
Procedure	173	0.29	45	1.59	218	0.34
Respiratory	54	0.09	118	4.17	172	0.27
Toxic effect	*	< 0.01	*	0.07	*	0.01
Mean maternal age +/− standard deviation	29.46 ± 6.07		29.67 ± 6.28		29.48 ± 6.08	

*Less than 10 patients.

### Exposure classification

#### Repurposed drug classification

**Figure 1:**
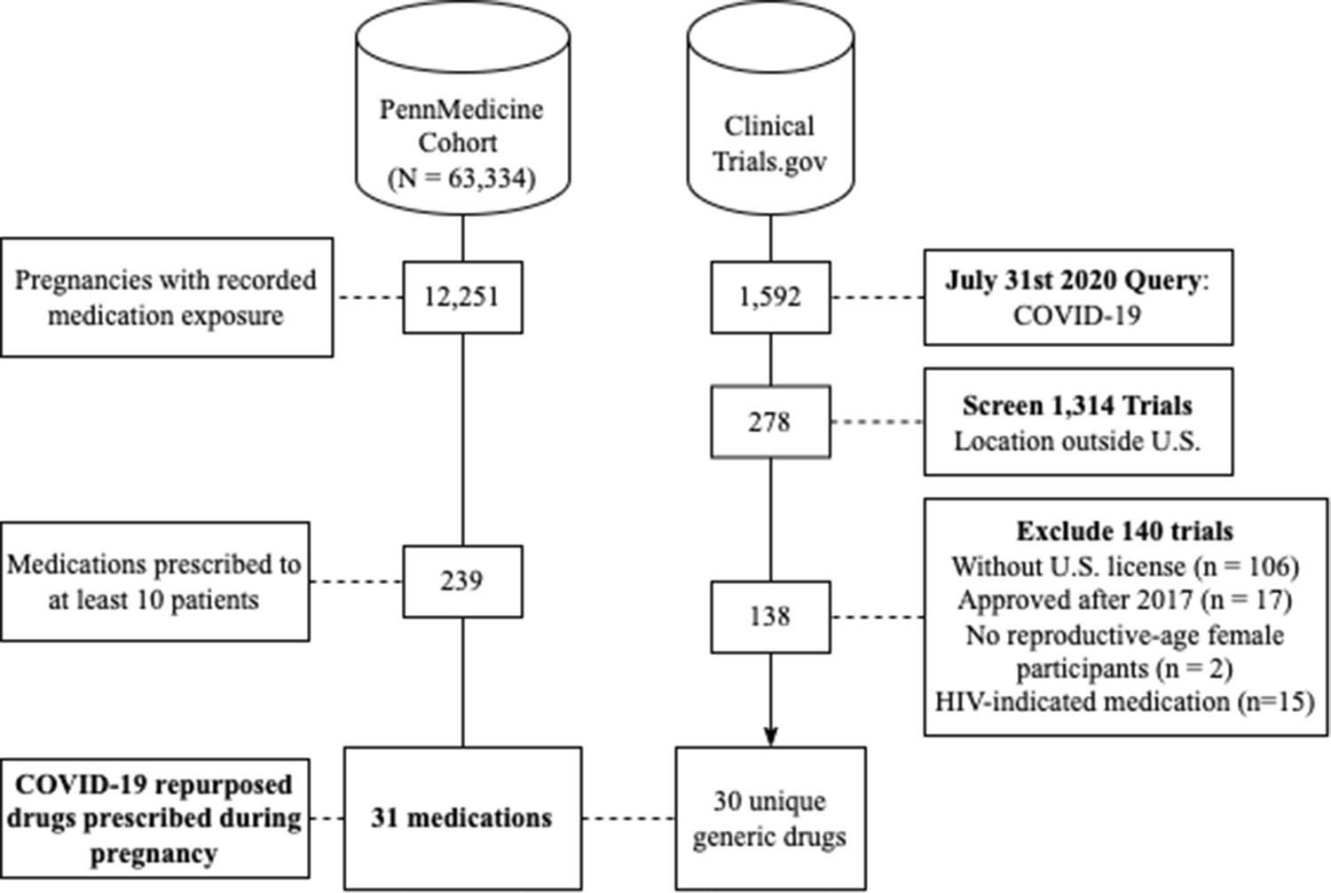
Flowchart of cohort and clinical trial selection process.

Our query of ClinicalTrials.gov returned 1,592 clinical trials focused on COVID-19 treatment. We excluded 1,071 trials from the query, including those: investigating drugs without US FDA approval and non-pharmacological interventions (n = 1037), investigating drugs FDA approved after 2017 (n = 18), investigating medications previously only indicated for HIV treatment (n = 15), and excluding reproductive age female participants (n = 16). After filtering based on this exclusion criteria, we identified 138 clinical trials, in which 30 generic medications were repurposed for COVID-19 treatment (See Fig. 1). See Table S4 in the supplementary materials for the 30 medications, their indications, proposed COVID-19 indications, and corresponding national clinical trial (NCT) identification numbers from our query.

#### Pregnancy exposure drug classification

Concerning the medications prescribed to our cohort, we manually annotated 239 medications with 45 broad classes of drugs. From this list, we identified 49 unique mediation names (31 unique generic medications) of drugs repurposed for the treatment of COVID-19 and its complications. Some medications included other active ingredients and therefore were not combined with the generic prescriptions. To combine generic and brand name equivalent drugs, we created 20 merged medication variables—resulting in 31 medication variables for analysis. We only included those medications having at least 10 pregnant patients exposed.

### Medication-wide association study of pregnancy outcomes

**Figure 2:**
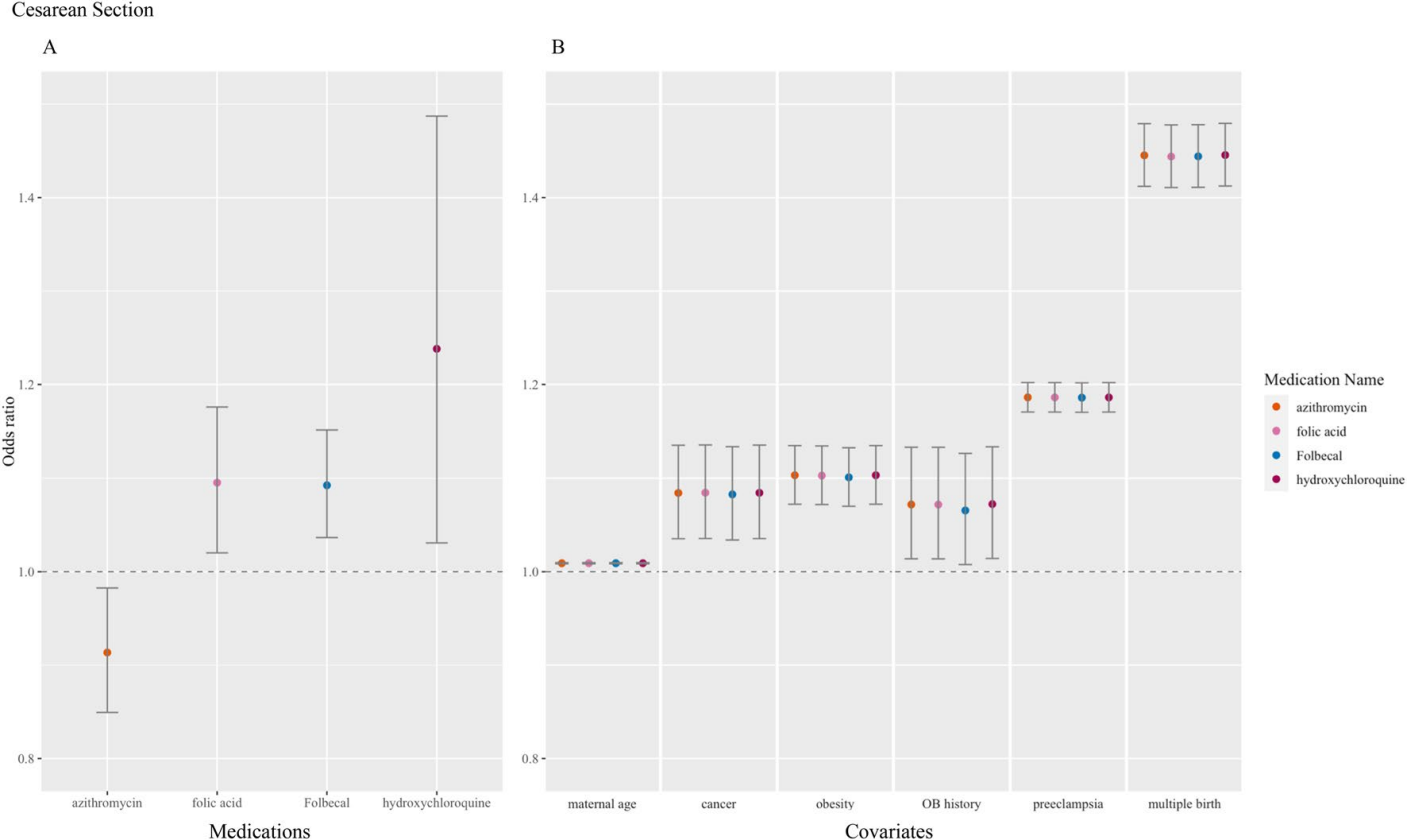
Medications and covariates significantly associated with cesarean section, odds ratio (95% confidence intervals). Plotted by (**A**) significant medications and (**B**) significant covariates.

**Figure 3:**
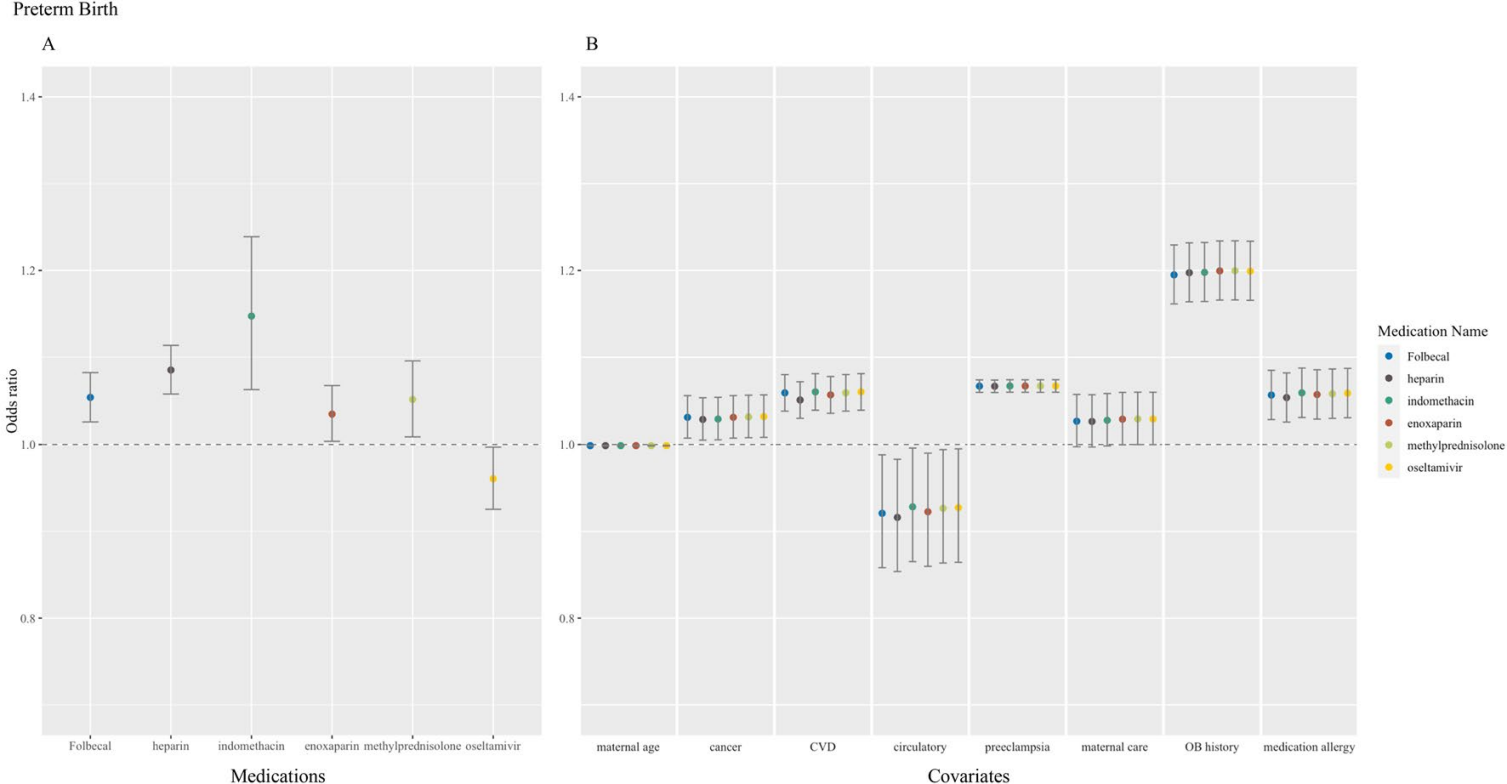
Medications and covariates significantly associated with preterm birth, odds ratio (95% confidence intervals). Plotted by (**A**) significant medications and (**B**) significant covariates.

**Figure 4:**
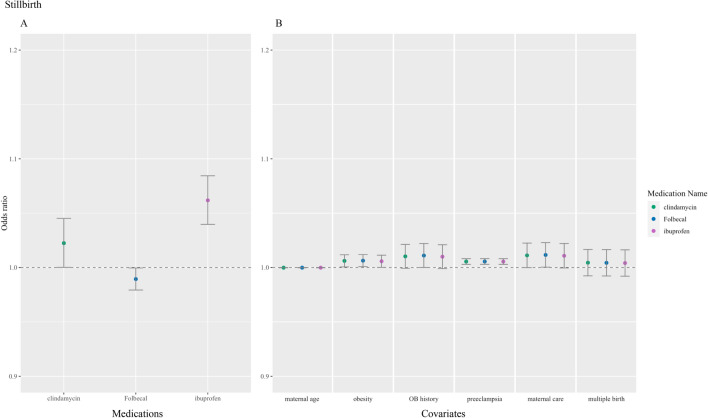
Medications and covariates significantly associated with stillbirth, odds ratio (95% confidence intervals). Plotted by (**A**) significant medications and (**B**) significant covariates.

The resulting model was adjusted for covariates associated with high risk (See Supplemental Table S2). Goodness-of-fit was determined by comparing adjusted and non-adjusted models; we report the values from the final adjusted models that consider patient comorbidities and other known risk factors for adverse pregnancy outcomes in Supplemental Table S5. The logistic regression model resulted in 11 medication names found significant (*P* < 0.05) for at least one pregnancy outcome, five of which were found significant with Bonferroni correction (see Table 2). The calculated odds ratio and 95% confidence intervals for the significant medications and covariates by pregnancy outcome: cesarean delivery (Fig. 2), preterm birth (Fig. 3) and stillbirth (Fig. 4). Notably, the covariates maternal age and preeclampsia diagnoses were found significantly associated (*P* < 0.05) across all three observed outcomes. Obstetric history diagnoses (i.e. personal history of complications of pregnancy) and cancer diagnoses were associated with cesarean section and preterm birth, while obesity diagnoses were associated with cesarean section and stillbirth. As expected, multiple birth was found highly associated with cesarean section delivery (see Fig. 2).

**Table 2:** Medications that potentially be repurposed for COVID-19 and their effects on pregnancy outcomes.

Drug/treatment class	Medication name^a^	Number exposed^b^	Odds ratio (95% CI)		
			Cesarean section	Preterm birth	Stillbirth
***Antivirals & antimalarials***					
Neuraminidase inhibitors	Oseltamivir	152	0.98 (0.91–1.06)	0.96 (0.93–1.00) *	0.99 (0.98–1.01)
Antiprotozoal agents	Mefloquine	18	0.88 (0.71–1.09)	0.95(0.85–1.06)	0.99 (0.95–1.04)
	Hydroxychloroquine	25	1.24 (1.03–1.49) *	1.04 (0.95–1.14)	0.99 (0.95–1.03)
***Antibiotics***					
Fluoroquinolone	Levofloxacin	13	1.05 (0.82–1.35)	1.03 (0.91–1.18)	0.99 (0.94–1.04)
Lincomycin	Clindamycin	64	0.99 (0.89–1.11)	1.03 (0.98–1.09)	1.02 (1.00–1.05)*
Macrolide	Azithromycin	154	0.91 (0.85–0.98) *	1.02 (0.98–1.06)	0.99 (0.98–1.01)
Penicillin	Amoxicillin	278	1.02 (0.97–1.08)	1.00 (0.97–1.03)	1.00 (0.99–1.01)
	Amoxicillin/clavulanate potassium	63	1.06 (0.94–1.19)	1.02 (0.96–1.08)	0.99 (0.97–1.01)
Sulfonamide	Sulfamethoxazole / trimethoprim	89	0.98 (0.89–1.08)	1.05 (1.00–1.10)	1.01 (0.99–1.03)
Tetracycline	Doxycycline	70	1.03 (0.93–1.15)	1.05 (1.00–1.11)	1.00 (0.98–1.02)
***Antidepressants***					
Select-serotonin reuptake inhibitors	Fluoxetine	33	1.08 (0.92–1.26)	1.05 (0.97–1.14)	0.99 (0.96–1.02)
***Antihistamines***					
Histamine-2 blockers	Famotidine	508	1.00 (0.97–1.05)	0.98 (0.96–1.00)	1.00 (0.99–1.00)
***Corticosteroids***					
Short-acting	Hydrocortisone	118	0.97 (0.89–1.06)	1.00 (0.96–1.04)	0.99 (0.98–1.01)
Intermediate-acting	Methylprednisolone	124	1.08 (1.00–1.17)	1.05 (1.01–1.10) *	1.00 (0.98–1.01)
	Prednisone	74	0.96 (0.86–1.07)	1.01 (0.96–1.07)	0.99 (0.97–1.01)
Long-acting	Dexamethasone	16	1.02 (0.82–1.28)	1.04 (0.93–1.17)	0.99 (0.95–1.04)
***Immunosuppressants***					
Calcineurin inhibitors	Tacrolimus	13	0.81 (0.62–1.07)	0.89 (0.78–1.03)	1.09 (1.03–1.15)**
**Medications for COVID-19 symptoms**					
***Supplements***					
	Folic acid	162	1.10 (1.02–1.18) **	1.00 (0.96–1.04)	0.99 (0.98–1.01)
	Calcium carbonate/folic acid/pyridoxine itamin B ^c^	309	1.09 (1.04–1.15) **	1.05 (1.03–1.08) **	0.99 (0.98–1.00)*
	Vitamin D	23	0.84 (0.69–1.01)	1.01 (0.92–1.11)	0.99 (0.96–1.03)
***Cardiovascular***					
Calcium channel blockers	Diltiazem	11	1.14 (0.87–1.49)	1.07 (0.93–1.23)	0.99 (0.94–1.04)
Beta blockers	Propranolol	11	0.77 (0.59–1.01)	0.96 (0.84–1.10)	0.99 (0.94–1.05)
***Coagulopathy***					
Anticoagulants	Enoxaparin	238	1.06 (0.99–1.12)	1.04 (1.00–1.07) *	1.00 (0.99–1.01)
	Heparin	345	1.00 (0.95–1.06)	1.09 (1.06–1.11) **	1.01 (1.00–1.02)*
***Pain***					
NSAIDs	Aspirin	136	1.05 (0.97–1.13)	1.03 (0.99–1.07)	1.00 (0.99–1.02)
	Ibuprofen	70	0.94 (0.84–1.04)	1.05 (0.99–1.11)	1.06 (1.04–1.08)**
	Indomethacin	36	1.02 (0.88–1.19)	1.15 (1.06–1.24) **	1.02 (0.99–1.05)
	Naproxen	27	0.86 (0.72–1.02)	0.98 (0.89–1.07)	1.03 (0.99–1.06)
***Respiratory***					
Bronchodilators	Budesonide	64	0.94 (0.84–1.05)	1.00 (0.94–1.05)	0.99 (0.97–1.01)
Leukotriene receptor antagonists	Montelukast	48	0.92 (0.81–1.05)	1.01 (0.95–1.08)	0.99 (0.97–1.02)
*Gastrointestinal*					
Proton pump inhibitors	Esomeprazole	41	1.05 (0.91–1.21)	0.99 (0.92–1.07)	0.99 (0.97–1.02)

^a^Medication names listed are generic names, although some patients were on brand name medications while others were on generic and these were merged based on common active ingredients.

^b^The number of pregnancies exposed to the generic medication during the observed prescription window (280 days before delivery up to 1 day before delivery). All pregnancies, exposed and none-exposed were included in the analysis.

^c^Medication contains other active ingredients.

*Nominal *P*-value <  = 0.05.

**Nominal *P*-value & *P*-value with Bonferroni adjustment <  = 0.05.

Concerning prescription medications, folic acid medications (folic acid and Folbecal) were inconsistently associated across all observed outcomes. Azithromycin (n = 154, odds ratio [OR] 0.91, 95% CI 0.85–0.98) and oseltamivir (n = 152, OR 0.96, 95% CI 0.93–1.00) were found conservatively protective of cesarean section and preterm birth, respectively. Two coagulopathy medications, heparin (n = 345, OR 1.09, 95% CI 1.06–1.11) and enoxaparin (n = 238, OR 1.04, 95% CI 1.00–1.07), were associated with preterm birth. Heparin was also associated with stillbirth (n = 345, OR 1.01, 95% CI 1.00–1.02). One of the three antiviral and antimalarial medications prescribed to our cohort, hydroxychloroquine (n = 25, OR 1.24, 95% CI 1.03–1.49), was found to be associated with cesarean section. One of the seven antibiotics prescribed, clindamycin, was associated with stillbirth in our analysis (n = 64, OR 1.02, 95% CI 1.00–1.05). Methylprednisolone, one of the four corticosteroids extracted, was associated with preterm birth (n = 124, OR 1.05, 95% CI 1.01–1.10). An immunosuppressant prescribed in our cohort, tacrolimus, was associated with stillbirth (n = 13, OR 1.09, 95% CI 1.03–1.15). Lastly, two of the four prescribed pain medications, ibuprofen (n = 70, OR 1.06, 95% CI 1.04–1.08) and indomethacin (n = 36, OR 1.15, 95% CI 1.06–1.24), were associated with stillbirth and preterm birth, respectively.

Overall, 10 generic medications repurposed in COVID-19 treatment were found significant *(P* < 0.05), and 21 of the generic medications sourced from our query were not associated with an increased risk of the observed adverse outcomes.

## Discussion

Our MWAS demonstrates the opportunity and challenges in pharmacovigilance using EHR data. With adjustment for comorbidities, 10 of the 31 medications identified in COVID-19 treatment clinical trials (5 of the 31 medications with Bonferroni adjustment) were found to be positively associated with at least one of the observed pregnancy outcomes. These associations warrant further investigation and model adjustment as necessary. More importantly, the other 21 medications with no positive association with adverse outcomes are potential candidates for the inclusion of pregnant patients in future clinical trials.

### Medications that aid with immune response

#### Antivirals and antimalarials

A recent retrospective cohort study suggests that hydroxychloroquine use in pregnancy does not increase or decrease obstetric or neonatal morbidities often associated with patients diagnosed with systemic lupus erythematosus^30^. We found a slight increase in risk for Cesarean delivery among our cohort, but no significant increases in preterm birth or stillbirth. Therefore, our findings suggest further caution than reported in prior literature with regard to hydroxychloroquine use in pregnancy.

Interestingly, in our study, we found that Oseltamivir had a slight negative association with preterm birth (i.e., was protective). Oseltamivir is an important antiviral therapy, with the potential to substantially mitigate increased risks of influenza in pregnancy. Therefore, osteltamivir might warrant further investigation as a drug that could be of use for pregnant people (also our sample size was relatively large with 152 patients exposed). For mefloquine, we found no statistically significant increases (or decreases) in any of the outcomes studied in this analysis.

#### Antibiotics

The macrolide antibiotic azithromycin was included in 19 clinical trials, often in combination with hydroxychloroquine. In our analysis, azithromycin was negatively associated with cesarean section. On the other hand, our results indicate an association of clindamycin with stillbirth. Both azithromycin and clindamycin are accepted as generally safe to use^31,32^. An estimated 10–25% of stillbirths may be caused by maternal or fetal infections, including bacterial infections^33^.

#### Steroids & immunosuppressants

Corticosteroids along with antibiotics (e.g., methylprednisolone and doxycycline) may be prescribed prior to embryo transfer in assisted reproductive therapy. Antenatal corticosteroids may also be prescribed to accelerate fetal lung maturation for those at risk for preterm labor^34^. Tacrolimus is an immunosuppressive medication that may be prescribed for post solid organ transplantation. While this population may be at an increased risk for prematurity, low birth weight, and cesarean section, our analysis found an association with stillbirth only^35,36^.

#### Supplements and host-directed therapies

The prenatal vitamin, Calcium carbonate / folic acid / pyridoxine / vitamin B12 (Folbecal), was positively associated with preterm birth and cesarean section, as well as negatively associated with stillbirth. Folic acid was found to be associated with cesarean section. The CDC recommends 400 mg daily dose of folic acid during pregnancy in prevention of neural tube defects. The supplement may be prescribed during the periconceptional period, when planning a pregnancy or when a previous pregnancy was affected by neural tube defects^37^. Current evidence shows the safety and preventative effects of folic acid during pregnancy^38^.

### Medications for COVID-19 symptoms

#### Anticoagulants and cardiovascular related medications

Heparin and enoxaparin, a low-molecular-weight heparin, were associated with preterm birth. Preeclampsia, as shown in Fig. 3B, is associated with preterm birth. A randomized controlled trial found the supplementation of enoxaparin in addition to standard high-risk care did not reduce the risk of recurrence of preeclampsia and small-for-gestational-age infants in subsequent pregnancies^39^.

#### Pain & anti-inflammatory therapies

Nonsteroidal anti-inflammatory drugs (NSAIDs) are among the most widely used drugs and are often used during pregnancy. Indomethacin has anti-inflammatory and antiviral properties, the latter of which was discovered in the wake of the SARS-CoV outbreak^40^. Our analysis found ibuprofen medications to be significantly associated with stillbirth, while indomethacin was associated with preterm birth. In October 2020, the FDA reported a Drug Safety Communication recommending avoiding the use of NSAIDs in pregnancy after 20 weeks of gestation because of risk of fetal renal dysfunction leading to oligohydramnios^41^. However, these associations may also reflect treatment in the prevention or recovery of these pregnancy outcomes. Indomethacin may be prescribed as a tocolytic agent in treatment of preterm labor^42^, as well as ibuprofen could be prescribed for stillbirth recovery.

#### Respiratory therapies

None of the three medications for respiratory therapies were found significantly associated with the observed pregnancy outcomes, in congruency with current knowledge^43^.

### Challenges in drug repurposing for COVID-19 treatment

We demonstrated that many clinical trials for COVID-19 treatment are underway, repurposing medications that patients in our cohort have been prescribed. Expedient design, small sample sizes, and lack of clinical outcome measures lead many studies to yield only preliminary evidence of a treatment’s safety and efficacy against COVID-19^44^. Randomized clinical trials often either do not include pregnant patients at all as study subjects or they include them only in a limited format^45^. The ethics regarding the inclusion of pregnant patients in clinical trials has been hotly debated in the past. However, the issue has moved to the forefront with the advent of a global pandemic^14,46,47^. Often pregnant patients with COVID-19 are only included in trials if they meet certain criteria, which vary by institution. For example, options for pregnant patients with severe COVID-19 were limited in some cases to the compassionate use of remdesivir or off-label drug use of hydroxychloroquine^46^.

New evidence regarding the pathophysiology, diagnosis, and treatment of the infection builds daily. The use of EHR data can accelerate research and provide justification on the inclusion and exclusion of pregnant individuals for clinical trials of repurposed drugs indicated for COVID-19 treatment and beyond. Thoughtfully including pregnant people in clinical research will lead to solid evidence for clinical care.

### Beyond COVID-19: need for and challenges of studies that include pregnant patients

Properly assessing the risks both to the mother and the fetus proves to be an obstacle in developing ethical frameworks for the inclusion of pregnant patients^48^. The purpose of our study was to develop a method to assess all drugs being considered at the clinical trial level to assess a.) was the drug ever prescribed to pregnant patients during pregnancy at our institution and b.) was there any evidence of increased risk to the mother or fetus after adjusting for other known comorbidities? Retrospective analysis can be the first step to rapidly screen medications that are less likely to cause harm to the mother and/or fetus. The medications we identified as to *not* increase risk could potentially be used in future clinical trials if pregnant patients were to be included. We recommend that for medications where harm was identified, additional study and evidence would be required before inclusion of pregnant patients in clinical trials.

Despite the best intentions to protect the pregnant population, the lack of drug safety knowledge is by design. The pregnant population is prescribed medication during pregnancy. Exclusion of the pregnant population should require thorough justification.

### Limitations and future work

Many factors influence how medications are processed in the body; our analysis does not account for dosage, route of administration, drug form, drug-to-drug interactions, or pharmacogenetics. For example, the two clinical trials including ibuprofen (NCT04382768, NCT04334629) are inhaled hypertonic ibuprofen and lipid ibuprofen, respectively. We did not investigate the medication route of administration (e.g., inhaled, oral) and therefore when we assessed ibuprofen it was all routes of administration (although a majority of administrations are likely to be oral and via tablet formulation). Other possible outcomes of interest (e.g. birthweight centile, congenital abnormalities) are beyond the scope of the EHR data available. Further work to connect the infant EHR data to the mother’s pregnancy data is necessary. We included prenatal medication exposure made during all pregnancy trimesters and did not subset to investigate trimester-specific effects. Sample size differs by medication prescribed (10 to 508 patients), which limits the power of our analysis. Due to a lack of precise information on the conception date, the defined medication exposure window in our analysis is constant across pregnancies. These constrained sample sizes and lack of conception dates led to the decision to not investigate trimester-specific effects. For pregnancy outcomes with a well-defined etiologically relevant window, this can provide more insight into the exposure-outcome associations.

We adjusted for diagnoses associated with maternal and fetal risk of the observed outcomes, within two years in order to address missingness common in EHR data. However, we do not have access to certain infection information, such as Human Immunodeficiency Virus status as part of this study. Furthermore, psychotropic medication prescription without a record of psychiatric diagnosis was observed in insurance claims data^49^. Therefore, the same may be true for EHR data, introducing missingness in the diagnoses we adjusted for in the study. Additionally, we are using prescription related information and therefore we cannot ascertain if the patient definitively took the prescribed medication. Our data is sourced from medication orders and does not indicate whether the prescription was filled, nor whether the medication was adhered to by the patient. This misclassification in our analysis may lead to overestimates in actual exposures, especially concerning outpatient prescriptions. External medications (e.g., over-the-counter medications, medication sharing with partners, and so forth) are outside the scope of EHR data and therefore these exposures are unaccounted for in our analysis. We recommend interpreting the results of over-the-counter medications (e.g. ibuprofen) with caution due to this factor. Future work includes incorporating deep phenotyping and machine learning techniques to improve understanding of drug exposure and pregnancy risk and the covariates that are most informative in this relationship^50^.

## Conclusion

The MWAS approach applied to EHR data in this study provides a framework that can be applied to all prescribed drugs and outcomes of interest for the purpose to assess the drug-outcome pairs. The true extent of the potential relationships requires further research and adjustments to the model. Our hypothesis-generating MWAS approach explores potential outcome-exposure relationships, which can be investigated in subsequent research. Our method allows researchers to bin candidate drugs into three categories: a.) those never used in pregnant women (and therefore excluded from this study); b.) those used in pregnant women that are associated with increased maternal or fetal risk and c.) those used in pregnant women without the increased maternal or fetal risk associated with them. The latter group of medications represents a set of drugs that shows potential for inclusion in future studies that may recruit pregnant patients in clinical trials. By understanding the known safety profiles of repurposed medications, our approach can promote the active intentional inclusion of pregnant patients in clinical trials in the future.

### Ethics statement

This study was approved by The University of Pennsylvania's Institutional Review Board.

## Supplementary Information

Supplementary Information 1.

Supplementary Information 2.

## Data Availability

This paper uses Electronic Health Record (EHR) data obtained from the Penn Medicine health system. The raw data is not freely available or shareable due to regulations involving patient privacy, per the HIPAA Privacy Rule that governs protected health information and therefore the ‘raw’ patient data is not shareable. We are sharing methods, results and supplementary information in this paper. Furthermore, code that can be used on similar patient data is available here: https://github.com/bolandlab/Davidson_PregnancyOutcomes_COVIDMeds along with fake patient data generated for the purposes of verifying our methods.

## Code availability

We are making our model code freely available at this GitHub website: https://github.com/bolandlab/Davidson_PregnancyOutcomes_COVIDMeds we will also be providing 'fake' synthetic patient data to test our code against for the purposes of verifying our models for reproducibility and research integrity. We are also providing information on the diagnosis codes used to identify patients as having various comorbidities, and outcomes.
