# Supplementary material for: A medication-wide association study (MWAS) on repurposed drugs for COVID-19 with Pre-pandemic prescription medication exposure and pregnancy outcomes
Source: Sci Rep. 2022 Nov 24;12:20314. doi: 10.1038/s41598-022-24218-1 (PMC9700703; doi:10.1038/s41598-022-24218-1)
Supplement: Supplementary file 2 — Supplementary Information 2. [file 41598_2022_24218_MOESM2_ESM.docx]

**Supplemental Tables for:**

**Davidson LM, Canelón SP, Boland MR.**

**A Medication-Wide Association Study (MWAS) on Repurposed Drugs for COVID-19 with Pre-Pandemic Prescription Medication Exposure and Pregnancy Outcomes**

**Table of Contents**

*Tables*

Table S1. ICD-9 and ICD-10 Codes Used to Identify Pregnancy Outcomes……………………..1

Table S2. Comorbidities for Adjustment in the Medication Wide Association Study…………..14

Table S3. ICD-9 and ICD-10 Codes Extracted For Adjustment in MWAS…………………..…15

Table S4. Medications Identified in COVID-19 Clinical Trials with Corresponding National Clinical Trial Codes……………………………………………………………...…….………….7

Table S5. Model Goodness of Fit Statistics for Final Models from the MWAS ………..………36

**Table 1.** ICD-9 and ICD-10 Codes Used to Identify Pregnancy Outcomes

| **Code** | **Pregnancy Outcome** | **ICD Version** | **Code Description** |
| --- | --- | --- | --- |
| 74 | Cesarean Section | ICD9 | Cesarean section and removal of fetus |
| 74 | Cesarean Section | ICD9 | Classical cesarean section |
| 74.1 | Cesarean Section | ICD9 | Low cervical cesarean section |
| 74.2 | Cesarean Section | ICD9 | Extraperitoneal cesarean section |
| 74.4 | Cesarean Section | ICD9 | Cesarean section of other specified type |
| 74.99 | Cesarean Section | ICD9 | Other cesarean section of unspecified type |
| 10D00Z0 | Cesarean Section | ICD10 | Extraction of Products of Conception, High, Open Approach |
| 10D00Z1 | Cesarean Section | ICD10 | Extraction of Products of Conception, Low, Open Approach |
| 10D00Z2 | Cesarean Section | ICD10 | Extraction of Products of Conception, Extraperitoneal, Open Approach |
| 669.7 | Cesarean Section | ICD9 | Cesarean delivery, without mention of indication, unspecified as to episode of care or not applicable |
| 669.71 | Cesarean Section | ICD9 | Cesarean delivery, without mention of indication, delivered, with or without mention of antepartum condition |
| 763.4 | Cesarean Section | ICD9 | Cesarean delivery affecting fetus or newborn |
| V30.01 | Cesarean Section | ICD9 | Single liveborn, born in hospital, delivered by cesarean section |
| V31.01 | Cesarean Section | ICD9 | Twin birth, mate liveborn, born in hospital, delivered by cesarean section |
| V32.01 | Cesarean Section | ICD9 | Twin birth, mate stillborn, born in hospital, delivered by cesarean section |
| V33.01 | Cesarean Section | ICD9 | Twin birth, unspecified whether mate liveborn or stillborn, born in hospital, delivered by cesarean section |
| V34.01 | Cesarean Section | ICD9 | Other multiple birth (three or more), mates all liveborn, born in hospital, delivered by cesarean section |
| V35.01 | Cesarean Section | ICD9 | Other multiple birth (three or more), mates all still born, born in hospital, delivered by cesarean section |
| V36.01 | Cesarean Section | ICD9 | Other multiple birth (three or more), mates liveborn and stillborn, born in hospital, delivered by cesarean section |
| V37.01 | Cesarean Section | ICD9 | Other multiple birth (three or more), unspecified whether mates liveborn or stillborn, born in hospital, delivered by cesarean section |
| V39.01 | Cesarean Section | ICD9 | Liveborn, unspecified whether single, twin or multiple, born in hospital, delivered by cesarean section |
| 649.81 | Cesarean Section | ICD9 | Onset (spontaneous) of labor after 37 completed weeks of gestation but before 39 completed weeks gestation, with delivery by (planned) cesarean section, delivered, with or without mention of antepartum condition |
| 649.82 | Cesarean Section | ICD9 | Onset (spontaneous) of labor after 37 completed weeks of gestation but before 39 completed weeks gestation, with delivery by (planned) cesarean section, delivered, with mention of postpartum complication |
| O75.82 | Cesarean Section | ICD10 | Onset (spontaneous) of labor after 37 completed weeks of gestation but before 39 completed weeks gestation, with delivery by (planned) cesarean section |
| O82 | Cesarean Section | ICD10 | Encounter for cesarean delivery without indication |
| Z38.01 | Cesarean Section | ICD10 | Single liveborn infant, delivered by cesarean |
| Z38.31 | Cesarean Section | ICD10 | Twin liveborn infant, delivered by cesarean |
| Z38.69 | Cesarean Section | ICD10 | Other multiple liveborn infant, delivered by cesarean |
| Z38.66 | Cesarean Section | ICD10 | Quintuplet liveborn infant, delivered by cesarean |
| Z38.62 | Cesarean Section | ICD10 | Triplet liveborn infant, delivered by cesarean |
| Z38.64 | Cesarean | ICD10 | Quadruplet liveborn infant, delivered by cesarean |
| 765.1 | Preterm birth | ICD9 | Other preterm infants, unspecified [weight] |
| 765.11 | Preterm birth | ICD9 | Other preterm infants, less than 500 grams |
| 765.12 | Preterm birth | ICD9 | Other preterm infants, 500-749 grams |
| 765.13 | Preterm birth | ICD9 | Other preterm infants, 750-999 grams |
| 765.14 | Preterm birth | ICD9 | Other preterm infants, 1,000-1,249 grams |
| 765.15 | Preterm birth | ICD9 | Other preterm infants, 1,250-1,499 grams |
| 765.16 | Preterm birth | ICD9 | Other preterm infants, 1,500-1,749 grams |
| 765.17 | Preterm birth | ICD9 | Other preterm infants, 1,750-1,999 grams |
| 765.18 | Preterm birth | ICD9 | Other preterm infants, 2,000-2,499 grams |
| 765.19 | Preterm birth | ICD9 | Other preterm infants, 2,500 grams and over |
| O60.1 | Preterm birth | ICD10 | Preterm labor with preterm delivery |
| O60.10 | Preterm birth | ICD10 | Preterm labor with preterm delivery, unspecified trimester |
| O60.10X0 | Preterm birth | ICD10 | Preterm labor with preterm delivery, unspecified trimester, not applicable or unspecified |
| O60.10X1 | Preterm birth | ICD10 | Preterm labor with preterm delivery, unspecified trimester, fetus 1 |
| O60.10X2 | Preterm birth | ICD10 | Preterm labor with preterm delivery, unspecified trimester, fetus 2 |
| O60.10X3 | Preterm birth | ICD10 | Preterm labor with preterm delivery, unspecified trimester, fetus 3 |
| O60.10X4 | Preterm birth | ICD10 | Preterm labor with preterm delivery, unspecified trimester, fetus 4 |
| O60.10X5 | Preterm birth | ICD10 | Preterm labor with preterm delivery, unspecified trimester, fetus 5 |
| O60.10X9 | Preterm birth | ICD10 | Preterm labor with preterm delivery, unspecified trimester, other fetus |
| O60.12 | Preterm birth | ICD10 | Preterm labor second trimester with preterm delivery second trimester |
| O60.12X0 | Preterm birth | ICD10 | Preterm labor second trimester with preterm delivery second trimester, not applicable or unspecified |
| O60.12X1 | Preterm birth | ICD10 | Preterm labor second trimester with preterm delivery second trimester, fetus 1 |
| O60.12X2 | Preterm birth | ICD10 | Preterm labor second trimester with preterm delivery second trimester, fetus 2 |
| O60.12X3 | Preterm birth | ICD10 | Preterm labor second trimester with preterm delivery second trimester, fetus 3 |
| O60.12X4 | Preterm birth | ICD10 | Preterm labor second trimester with preterm delivery second trimester, fetus 4 |
| O60.12X5 | Preterm birth | ICD10 | Preterm labor second trimester with preterm delivery second trimester, fetus 5 |
| O60.12X9 | Preterm birth | ICD10 | Preterm labor second trimester with preterm delivery second trimester, other fetus |
| O60.13 | Preterm birth | ICD10 | Preterm labor second trimester with preterm delivery third trimester |
| O60.13X0 | Preterm birth | ICD10 | Preterm labor second trimester with preterm delivery third trimester, not applicable or unspecified |
| O60.13X1 | Preterm birth | ICD10 | Preterm labor second trimester with preterm delivery third trimester, fetus 1 |
| O60.13X2 | Preterm birth | ICD10 | Preterm labor second trimester with preterm delivery third trimester, fetus 2 |
| O60.13X3 | Preterm birth | ICD10 | Preterm labor second trimester with preterm delivery third trimester, fetus 3 |
| O60.13X4 | Preterm birth | ICD10 | Preterm labor second trimester with preterm delivery third trimester, fetus 4 |
| O60.13X5 | Preterm birth | ICD10 | Preterm labor second trimester with preterm delivery third trimester, fetus 5 |
| O60.13X9 | Preterm birth | ICD10 | Preterm labor second trimester with preterm delivery third trimester, other fetus |
| O60.14 | Preterm birth | ICD10 | Preterm labor third trimester with preterm delivery third trimester |
| O60.14X0 | Preterm birth | ICD10 | Preterm labor third trimester with preterm delivery third trimester, not applicable or unspecified |
| O60.14X1 | Preterm birth | ICD10 | Preterm labor third trimester with preterm delivery third trimester, fetus 1 |
| O60.14X2 | Preterm birth | ICD10 | Preterm labor third trimester with preterm delivery third trimester, fetus 2 |
| O60.14X3 | Preterm birth | ICD10 | Preterm labor third trimester with preterm delivery third trimester, fetus 3 |
| O60.14X4 | Preterm birth | ICD10 | Preterm labor third trimester with preterm delivery third trimester, fetus 4 |
| O60.14X5 | Preterm birth | ICD10 | Preterm labor third trimester with preterm delivery third trimester, fetus 5 |
| O60.14X9 | Preterm birth | ICD10 | Preterm labor third trimester with preterm delivery third trimester, other fetus |
| P07.2 | Preterm birth | ICD10 | Extreme immaturity of newborn |
| P07.20 | Preterm birth | ICD10 | Extreme immaturity of newborn, unspecified weeks of gestation |
| P07.21 | Preterm birth | ICD10 | Extreme immaturity of newborn, gestational age less than 23 completed weeks |
| P07.22 | Preterm birth | ICD10 | Extreme immaturity of newborn, gestational age 23 completed weeks |
| P07.23 | Preterm birth | ICD10 | Extreme immaturity of newborn, gestational age 24 completed weeks |
| P07.24 | Preterm birth | ICD10 | Extreme immaturity of newborn, gestational age 25 completed weeks |
| P07.25 | Preterm birth | ICD10 | Extreme immaturity of newborn, gestational age 26 completed weeks |
| P07.26 | Preterm birth | ICD10 | Extreme immaturity of newborn, gestational age 27 completed weeks |
| P07.3 | Preterm birth | ICD10 | Preterm [premature] newborn [other] |
| P07.30 | Preterm birth | ICD10 | Preterm newborn, unspecified weeks of gestation |
| P07.31 | Preterm birth | ICD10 | Preterm newborn, gestational age 28 completed weeks |
| P07.32 | Preterm birth | ICD10 | Preterm newborn, gestational age 29 completed weeks |
| P07.33 | Preterm birth | ICD10 | Preterm newborn, gestational age 30 completed weeks |
| P07.34 | Preterm birth | ICD10 | Preterm newborn, gestational age 31 completed weeks |
| P07.35 | Preterm birth | ICD10 | Preterm newborn, gestational age 32 completed weeks |
| P07.36 | Preterm birth | ICD10 | Preterm newborn, gestational age 33 completed weeks |
| P07.37 | Preterm birth | ICD10 | Preterm newborn, gestational age 34 completed weeks |
| P07.38 | Preterm birth | ICD10 | Preterm newborn, gestational age 35 completed weeks |
| P07.39 | Preterm birth | ICD10 | Preterm newborn, gestational age 36 completed weeks |
| V27.1 | Stillbirth | ICD9 | Outcome of delivery, single stillborn |
| V27.3 | Stillbirth | ICD9 | Outcome of delivery, twins, one liveborn and one stillborn |
| V27.4 | Stillbirth | ICD9 | Outcome of delivery, twins, both stillborn |
| V27.7 | Stillbirth | ICD9 | Outcome of delivery, other multiple birth, all stillborn |
| V32.00 | Stillbirth | ICD9 | Twin birth, mate stillborn, born in hospital, delivered without mention of cesarean section |
| V32.01 | Stillbirth | ICD9 | Twin birth, mate stillborn, born in hospital, delivered by cesarean section |
| V33.00 | Stillbirth | ICD9 | Twin birth, unspecified whether mate liveborn or stillborn, born in hospital, delivered without mention of cesarean section |
| V33.01 | Stillbirth | ICD9 | Twin birth, unspecified whether mate liveborn or stillborn, born in hospital, delivered by cesarean section |
| V35.00 | Stillbirth | ICD9 | Other multiple birth (three or more), mates all still born, born in hospital, delivered without mention of cesarean section |
| V35.01 | Stillbirth | ICD9 | Other multiple birth (three or more), mates all still born, born in hospital, delivered by cesarean section |
| V36.00 | Stillbirth | ICD9 | Other multiple birth (three or more), mates liveborn and stillborn, born in hospital, delivered without mention of cesarean section |
| V36.01 | Stillbirth | ICD9 | Other multiple birth (three or more), mates liveborn and stillborn, born in hospital, delivered by cesarean section |
| V37.00 | Stillbirth | ICD9 | Other multiple birth (three or more), unspecified whether mates liveborn or stillborn, born in hospital, delivered without mention of cesarean section |
| V37.01 | Stillbirth | ICD9 | Other multiple birth (three or more), unspecified whether mates liveborn or stillborn, born in hospital, delivered by cesarean section |
| Z37.3 | Stillbirth | ICD10 | Twins, one liveborn and one stillborn |
| Z37.4 | Stillbirth | ICD10 | Twins, both stillborn |
| Z37.6 | Stillbirth | ICD10 | Other multiple births, some liveborn |
| Z37.60 | Stillbirth | ICD10 | Multiple births, unspecified, some liveborn |
| Z37.61 | Stillbirth | ICD10 | Triplets, some liveborn |
| Z37.62 | Stillbirth | ICD10 | Quadruplets, some liveborn |
| Z37.63 | Stillbirth | ICD10 | Quintuplets, some liveborn |
| Z37.64 | Stillbirth | ICD10 | Sextuplets, some liveborn |
| Z37.69 | Stillbirth | ICD10 | Other multiple births, some liveborn |
| Z37.7 | Stillbirth | ICD10 | Other multiple births, all stillborn |

**Table S2. Comorbidities for Adjustment in the Medication Wide Association Study.**

| **Comorbidities for Adjustment in Medication Wide Association Study ^a^** | | **Association(s)** |
| --- | --- | --- |
| **Communicable Disease** | |  |
|  | Infectious Disease | infertility & reproductive health |
| **Non-Communicable Disease** | |  |
|  | Autoimmune Disease | high risk pregnancy |
|  | Cancer | high risk pregnancy, reproductive health |
|  | Cardiovascular Disease | high risk pregnancy |
|  | Cerebrovascular Disease | high risk pregnancy |
|  | Circulatory Disease | high risk pregnancy |
|  | Respiratory Disease | high risk pregnancy |
| **Medical Status** | |  |
|  | Adverse Drug | high risk pregnancy, prescription to alternative medication |
|  | Maternal Age | high risk pregnancy, reproductive health |
|  | Maternal Care | high risk pregnancy, reproductive health |
|  | Multiple Birth | high risk pregnancy |
|  | Obesity | high risk pregnancy |
|  | Organ Transplant / Acquired Absence | high risk pregnancy |
|  | Preeclampsia | high risk pregnancy |
|  | Procedure | pain medication prescription |
|  | Toxic Effect | toxic agent exposure, reproductive health |
| **Medical History** | |  |
|  | Medication Allergy | autoimmune disease, prescription to alternative medication |
|  | Obstetric History | personal history of obstetric complication |

^a^ ICD9/ICD10 diagnoses from 2 years prior until 1 day prior to estimated delivery date.

**Table S3. ICD-9 and ICD-10 codes used to identify comorbidities for adjustment in MWAS**

| CODE | DISEASE_GROUP | CODE_DESCRIPTION |
| --- | --- | --- |
| 965.4 | adverse drug effect | POISONING BY AROMATIC ANALGESICS, NOT ELSEWHERE CLASSIFIED |
| 969.4 | adverse drug effect | POISONING BY BENZODIAZEPINE-BASED TRANQUILIZERS |
| T44.8X6A | adverse drug effect | UNDERDOSING OF CENTRALLY-ACTING AND ADRENERGIC-NEURON-BLOCKING AGENTS, INITIAL ENCOUNTER |
| 970.81 | adverse drug effect | POISONING BY COCAINE |
| 965.61 | adverse drug effect | POISONING BY PROPIONIC ACID DERIVATIVES |
| T38.0X5A | adverse drug effect | ADVERSE EFFECT OF GLUCOCORTICOIDS AND SYNTHETIC ANALOGUES, INITIAL ENCOUNTER |
| T47.4X5A | adverse drug effect | ADVERSE EFFECT OF OTHER LAXATIVES, INITIAL ENCOUNTER |
| T45.0X5A | adverse drug effect | ADVERSE EFFECT OF ANTIALLERGIC AND ANTIEMETIC DRUGS, INITIAL ENCOUNTER |
| T38.2X5A | adverse drug effect | ADVERSE EFFECT OF ANTITHYROID DRUGS, INITIAL ENCOUNTER |
| T38.3X5A | adverse drug effect | ADVERSE EFFECT OF INSULIN AND ORAL HYPOGLYCEMIC [ANTIDIABETIC] DRUGS, INITIAL ENCOUNTER |
| 969 | adverse drug effect | POISONING BY ANTIDEPRESSANT, UNSPECIFIED |
| 977.8 | adverse drug effect | POISONING BY OTHER SPECIFIED DRUGS AND MEDICINAL SUBSTANCES |
| T43.595A | adverse drug effect | ADVERSE EFFECT OF OTHER ANTIPSYCHOTICS AND NEUROLEPTICS, INITIAL ENCOUNTER |
| T46.1X5A | adverse drug effect | ADVERSE EFFECT OF CALCIUM-CHANNEL BLOCKERS, INITIAL ENCOUNTER |
| 966.1 | adverse drug effect | POISONING BY HYDANTOIN DERIVATIVES |
| T40.605A | adverse drug effect | ADVERSE EFFECT OF UNSPECIFIED NARCOTICS, INITIAL ENCOUNTER |
| 965.09 | adverse drug effect | POISONING BY OTHER OPIATES AND RELATED NARCOTICS |
| 966.3 | adverse drug effect | POISONING BY OTHER AND UNSPECIFIED ANTICONVULSANTS |
| T37.5X6A | adverse drug effect | UNDERDOSING OF ANTIVIRAL DRUGS, INITIAL ENCOUNTER |
| T39.1X2A | adverse drug effect | POISONING BY 4-AMINOPHENOL DERIVATIVES, INTENTIONAL SELF-HARM, INITIAL ENCOUNTER |
| T40.5X1A | adverse drug effect | POISONING BY COCAINE, ACCIDENTAL (UNINTENTIONAL), INITIAL ENCOUNTER |
| T36.1X5A | adverse drug effect | ADVERSE EFFECT OF CEPHALOSPORINS AND OTHER BETA-LACTAM ANTIBIOTICS, INITIAL ENCOUNTER |
| T36.8X5A | adverse drug effect | ADVERSE EFFECT OF OTHER SYSTEMIC ANTIBIOTICS, INITIAL ENCOUNTER |
| T45.515A | adverse drug effect | ADVERSE EFFECT OF ANTICOAGULANTS, INITIAL ENCOUNTER |
| T40.2X5A | adverse drug effect | ADVERSE EFFECT OF OTHER OPIOIDS, INITIAL ENCOUNTER |
| T40.4X5A | adverse drug effect | ADVERSE EFFECT OF OTHER SYNTHETIC NARCOTICS, INITIAL ENCOUNTER |
| T42.6X5A | adverse drug effect | ADVERSE EFFECT OF OTHER ANTIEPILEPTIC AND SEDATIVE-HYPNOTIC DRUGS, INITIAL ENCOUNTER |
| T42.75XA | adverse drug effect | ADVERSE EFFECT OF UNSPECIFIED ANTIEPILEPTIC AND SEDATIVE-HYPNOTIC DRUGS, INITIAL ENCOUNTER |
| 710 | autoimmune | SYSTEMIC LUPUS ERYTHEMATOSUS |
| 279.49 | autoimmune | AUTOIMMUNE DISEASE, NOT ELSEWHERE CLASSIFIED |
| 279.49 | autoimmune | AUTOIMMUNE DISEASE, NOT ELSEWHERE CLASSIFIED |
| D86.9 | autoimmune | SARCOIDOSIS, UNSPECIFIED |
| D86.3 | autoimmune | SARCOIDOSIS OF SKIN |
| D68.61 | autoimmune | ANTIPHOSPHOLIPID SYNDROME |
| M32.9 | autoimmune | SYSTEMIC LUPUS ERYTHEMATOSUS, UNSPECIFIED |
| 571.42 | autoimmune | AUTOIMMUNE HEPATITIS |
| D89.0 | autoimmune | POLYCLONAL HYPERGAMMAGLOBULINEMIA |
| 695.4 | autoimmune | LUPUS ERYTHEMATOSUS |
| E06.3 | autoimmune | AUTOIMMUNE THYROIDITIS |
| 279 | autoimmune | HYPOGAMMAGLOBULINEMIA, UNSPECIFIED |
| D68.62 | autoimmune | LUPUS ANTICOAGULANT SYNDROME |
| 218.9 | cancer | LEIOMYOMA OF UTERUS, UNSPECIFIED |
| 218.1 | cancer | INTRAMURAL LEIOMYOMA OF UTERUS |
| 201.9 | cancer | HODGKINS DISEASE, UNSPECIFIED TYPE, UNSPECIFIED SITE, EXTRANODAL AND SOLID ORGAN SITES |
| 236.2 | cancer | NEOPLASM OF UNCERTAIN BEHAVIOR OF OVARY |
| V10.43 | cancer | PERSONAL HISTORY OF MALIGNANT NEOPLASM OF OVARY |
| 220 | cancer | BENIGN NEOPLASM OF OVARY |
| V10.89 | cancer | PERSONAL HISTORY OF MALIGNANT NEOPLASM OF OTHER SITES |
| 196 | cancer | SECONDARY AND UNSPECIFIED MALIGNANT NEOPLASM OF LYMPH NODES OF HEAD, FACE, AND NECK |
| V10.22 | cancer | PERSONAL HISTORY OF MALIGNANT NEOPLASM OF NASAL CAVITIES, MIDDLE EAR, AND ACCESSORY SINUSES |
| 238.71 | cancer | ESSENTIAL THROMBOCYTHEMIA |
| 218 | cancer | SUBMUCOUS LEIOMYOMA OF UTERUS |
| 237.71 | cancer | NEUROFIBROMATOSIS, TYPE 1 [VON RECKLINGHAUSENS DISEASE] |
| 228.09 | cancer | HEMANGIOMA OF OTHER SITES |
| 193 | cancer | MALIGNANT NEOPLASM OF THYROID GLAND |
| 198.89 | cancer | SECONDARY MALIGNANT NEOPLASM OF OTHER SPECIFIED SITES |
| V10.81 | cancer | PERSONAL HISTORY OF MALIGNANT NEOPLASM OF BONE |
| V10.3 | cancer | PERSONAL HISTORY OF MALIGNANT NEOPLASM OF BREAST |
| 237.7 | cancer | NEUROFIBROMATOSIS, UNSPECIFIED |
| D27.0 | cancer | BENIGN NEOPLASM OF RIGHT OVARY |
| 188.9 | cancer | MALIGNANT NEOPLASM OF BLADDER, PART UNSPECIFIED |
| 215.5 | cancer | OTHER BENIGN NEOPLASM OF CONNECTIVE AND OTHER SOFT TISSUE OF ABDOMEN |
| 238.1 | cancer | NEOPLASM OF UNCERTAIN BEHAVIOR OF CONNECTIVE AND OTHER SOFT TISSUE |
| 197 | cancer | SECONDARY MALIGNANT NEOPLASM OF LUNG |
| 228 | cancer | HEMANGIOMA OF UNSPECIFIED SITE |
| D20.1 | cancer | BENIGN NEOPLASM OF SOFT TISSUE OF PERITONEUM |
| D25.2 | cancer | SUBSEROSAL LEIOMYOMA OF UTERUS |
| D42.0 | cancer | NEOPLASM OF UNCERTAIN BEHAVIOR OF CEREBRAL MENINGES |
| C71.9 | cancer | MALIGNANT NEOPLASM OF BRAIN, UNSPECIFIED |
| Z92.21 | cancer | PERSONAL HISTORY OF ANTINEOPLASTIC CHEMOTHERAPY |
| D25.1 | cancer | INTRAMURAL LEIOMYOMA OF UTERUS |
| 229.9 | cancer | BENIGN NEOPLASM OF UNSPECIFIED SITE |
| V10.87 | cancer | PERSONAL HISTORY OF MALIGNANT NEOPLASM OF THYROID |
| 211.6 | cancer | BENIGN NEOPLASM OF PANCREAS, EXCEPT ISLETS OF LANGERHANS |
| Z85.3 | cancer | PERSONAL HISTORY OF MALIGNANT NEOPLASM OF BREAST |
| 227.3 | cancer | BENIGN NEOPLASM OF PITUITARY GLAND AND CRANIOPHARYNGEAL DUCT |
| 211.5 | cancer | BENIGN NEOPLASM OF LIVER AND BILIARY PASSAGES |
| D17.71 | cancer | BENIGN LIPOMATOUS NEOPLASM OF KIDNEY |
| 210.2 | cancer | BENIGN NEOPLASM OF MAJOR SALIVARY GLANDS |
| V87.41 | cancer | PERSONAL HISTORY OF ANTINEOPLASTIC CHEMOTHERAPY |
| 218.2 | cancer | SUBSEROUS LEIOMYOMA OF UTERUS |
| D25.9 | cancer | LEIOMYOMA OF UTERUS, UNSPECIFIED |
| 213.2 | cancer | BENIGN NEOPLASM OF VERTEBRAL COLUMN, EXCLUDING SACRUM AND COCCYX |
| 202.8 | cancer | OTHER MALIGNANT LYMPHOMAS, UNSPECIFIED SITE, EXTRANODAL AND SOLID ORGAN SITES |
| 217 | cancer | BENIGN NEOPLASM OF BREAST |
| V10.82 | cancer | PERSONAL HISTORY OF MALIGNANT MELANOMA OF SKIN |
| V10.88 | cancer | PERSONAL HISTORY OF MALIGNANT NEOPLASM OF OTHER ENDOCRINE GLANDS AND RELATED STRUCTURES |
| 180 | cancer | MALIGNANT NEOPLASM OF ENDOCERVIX |
| V10.41 | cancer | PERSONAL HISTORY OF MALIGNANT NEOPLASM OF CERVIX UTERI |
| 214.8 | cancer | LIPOMA OF OTHER SPECIFIED SITES |
| 198.6 | cancer | SECONDARY MALIGNANT NEOPLASM OF OVARY |
| 159.9 | cancer | MALIGNANT NEOPLASM OF ILL-DEFINED SITES WITHIN THE DIGESTIVE ORGANS AND PERITONEUM |
| V10.83 | cancer | PERSONAL HISTORY OF OTHER MALIGNANT NEOPLASM OF SKIN |
| C71.3 | cancer | MALIGNANT NEOPLASM OF PARIETAL LOBE |
| O9A.112 | cancer | MALIGNANT NEOPLASM COMPLICATING PREGNANCY, SECOND TRIMESTER |
| V10.21 | cancer | PERSONAL HISTORY OF MALIGNANT NEOPLASM OF LARYNX |
| 236 | cancer | NEOPLASM OF UNCERTAIN BEHAVIOR OF UTERUS |
| 225.2 | cancer | BENIGN NEOPLASM OF CEREBRAL MENINGES |
| 201.91 | cancer | HODGKINS DISEASE, UNSPECIFIED TYPE, LYMPH NODES OF HEAD, FACE, AND NECK |
| 201.51 | cancer | HODGKINS DISEASE, NODULAR SCLEROSIS, LYMPH NODES OF HEAD, FACE, AND NECK |
| 174.9 | cancer | MALIGNANT NEOPLASM OF BREAST (FEMALE), UNSPECIFIED |
| 172.7 | cancer | MALIGNANT MELANOMA OF SKIN OF LOWER LIMB, INCLUDING HIP |
| 196.5 | cancer | SECONDARY AND UNSPECIFIED MALIGNANT NEOPLASM OF LYMPH NODES OF INGUINAL REGION AND LOWER LIMB |
| 172.6 | cancer | MALIGNANT MELANOMA OF SKIN OF UPPER LIMB, INCLUDING SHOULDER |
| V10.02 | cancer | PERSONAL HISTORY OF MALIGNANT NEOPLASM OF OTHER AND UNSPECIFIED ORAL CAVITY AND PHARYNX |
| 221 | cancer | BENIGN NEOPLASM OF FALLOPIAN TUBE AND UTERINE LIGAMENTS |
| V10.05 | cancer | PERSONAL HISTORY OF MALIGNANT NEOPLASM OF LARGE INTESTINE |
| V10.62 | cancer | PERSONAL HISTORY OF MYELOID LEUKEMIA |
| C73 | cancer | MALIGNANT NEOPLASM OF THYROID GLAND |
| 173.91 | cancer | BASAL CELL CARCINOMA OF SKIN, SITE UNSPECIFIED |
| C44.91 | cancer | BASAL CELL CARCINOMA OF SKIN, UNSPECIFIED |
| 228.1 | cancer | LYMPHANGIOMA, ANY SITE |
| 170 | cancer | MALIGNANT NEOPLASM OF BONES OF SKULL AND FACE, EXCEPT MANDIBLE |
| D24.9 | cancer | BENIGN NEOPLASM OF UNSPECIFIED BREAST |
| 238.79 | cancer | OTHER LYMPHATIC AND HEMATOPOIETIC TISSUES |
| 227 | cancer | BENIGN NEOPLASM OF ADRENAL GLAND |
| 237.5 | cancer | NEOPLASM OF UNCERTAIN BEHAVIOR OF BRAIN AND SPINAL CORD |
| 170.7 | cancer | MALIGNANT NEOPLASM OF LONG BONES OF LOWER LIMB |
| 233.1 | cancer | CARCINOMA IN SITU OF CERVIX UTERI |
| 228.04 | cancer | HEMANGIOMA OF INTRA-ABDOMINAL STRUCTURES |
| Z85.07 | cancer | PERSONAL HISTORY OF MALIGNANT NEOPLASM OF PANCREAS |
| V10.90 | cancer | PERSONAL HISTORY OF UNSPECIFIED MALIGNANT NEOPLASM |
| 211.3 | cancer | BENIGN NEOPLASM OF COLON |
| D27.9 | cancer | BENIGN NEOPLASM OF UNSPECIFIED OVARY |
| V10.72 | cancer | PERSONAL HISTORY OF HODGKINS DISEASE |
| 215.9 | cancer | OTHER BENIGN NEOPLASM OF CONNECTIVE AND OTHER SOFT TISSUE, SITE UNSPECIFIED |
| D27.1 | cancer | BENIGN NEOPLASM OF LEFT OVARY |
| 205.1 | cancer | CHRONIC MYELOID LEUKEMIA, WITHOUT MENTION OF HAVING ACHIEVED REMISSION |
| C50.911 | cancer | MALIGNANT NEOPLASM OF UNSPECIFIED SITE OF RIGHT FEMALE BREAST |
| C91.11 | cancer | CHRONIC LYMPHOCYTIC LEUKEMIA OF B-CELL TYPE IN REMISSION |
| C91.01 | cancer | ACUTE LYMPHOBLASTIC LEUKEMIA, IN REMISSION |
| O9A.113 | cancer | MALIGNANT NEOPLASM COMPLICATING PREGNANCY, THIRD TRIMESTER |
| Z51.11 | cancer | ENCOUNTER FOR ANTINEOPLASTIC CHEMOTHERAPY |
| C11.9 | cancer | MALIGNANT NEOPLASM OF NASOPHARYNX, UNSPECIFIED |
| 191.9 | cancer | MALIGNANT NEOPLASM OF BRAIN, UNSPECIFIED |
| 233 | cancer | CARCINOMA IN SITU OF BREAST |
| 196.3 | cancer | SECONDARY AND UNSPECIFIED MALIGNANT NEOPLASM OF LYMPH NODES OF AXILLA AND UPPER LIMB |
| 215 | cancer | OTHER BENIGN NEOPLASM OF CONNECTIVE AND OTHER SOFT TISSUE OF HEAD, FACE, AND NECK |
| D35.2 | cancer | BENIGN NEOPLASM OF PITUITARY GLAND |
| C79.51 | cancer | SECONDARY MALIGNANT NEOPLASM OF BONE |
| 174.1 | cancer | MALIGNANT NEOPLASM OF CENTRAL PORTION OF FEMALE BREAST |
| 212.9 | cancer | BENIGN NEOPLASM OF RESPIRATORY AND INTRATHORACIC ORGANS, SITE UNSPECIFIED |
| 238.4 | cancer | POLYCYTHEMIA VERA |
| 171.3 | cancer | MALIGNANT NEOPLASM OF CONNECTIVE AND OTHER SOFT TISSUE OF LOWER LIMB, INCLUDING HIP |
| C05.0 | cancer | MALIGNANT NEOPLASM OF HARD PALATE |
| V10.42 | cancer | PERSONAL HISTORY OF MALIGNANT NEOPLASM OF OTHER PARTS OF UTERUS |
| V10.44 | cancer | PERSONAL HISTORY OF MALIGNANT NEOPLASM OF OTHER FEMALE GENITAL ORGANS |
| 226 | cancer | BENIGN NEOPLASM OF THYROID GLANDS |
| 426.82 | cardiovascular | LONG QT SYNDROME |
| 401.9 | cardiovascular | UNSPECIFIED ESSENTIAL HYPERTENSION |
| 427.89 | cardiovascular | OTHER SPECIFIED CARDIAC DYSRHYTHMIAS |
| 427.61 | cardiovascular | SUPRAVENTRICULAR PREMATURE BEATS |
| V12.51 | cardiovascular | PERSONAL HISTORY OF VENOUS THROMBOSIS AND EMBOLISM |
| 405.99 | cardiovascular | OTHER UNSPECIFIED SECONDARY HYPERTENSION |
| 429.9 | cardiovascular | HEART DISEASE, UNSPECIFIED |
| 424.3 | cardiovascular | PULMONARY VALVE DISORDERS |
| 415.19 | cardiovascular | OTHER PULMONARY EMBOLISM AND INFARCTION |
| V12.55 | cardiovascular | HX PULMONARY EMBOLISM |
| 424 | cardiovascular | MITRAL VALVE DISORDERS |
| 427.31 | cardiovascular | ATRIAL FIBRILLATION |
| 429.3 | cardiovascular | CARDIOMEGALY |
| I34.0 | cardiovascular | NONRHEUMATIC MITRAL (VALVE) INSUFFICIENCY |
| I48.0 | cardiovascular | PAROXYSMAL ATRIAL FIBRILLATION |
| I26.99 | cardiovascular | OTHER PULMONARY EMBOLISM WITHOUT ACUTE COR PULMONALE |
| Z86.73 | cardiovascular | PERSONAL HISTORY OF TRANSIENT ISCHEMIC ATTACK (TIA), AND CEREBRAL INFARCTION WITHOUT RESIDUAL DEFICITS |
| Z86.711 | cardiovascular | PERSONAL HISTORY OF PULMONARY EMBOLISM |
| I25.2 | cardiovascular | OLD MYOCARDIAL INFARCTION |
| I42.9 | cardiovascular | CARDIOMYOPATHY, UNSPECIFIED |
| 426.4 | cardiovascular | RIGHT BUNDLE BRANCH BLOCK |
| 428 | cardiovascular | CONGESTIVE HEART FAILURE, UNSPECIFIED |
| 425.4 | cardiovascular | OTHER PRIMARY CARDIOMYOPATHIES |
| 428.23 | cardiovascular | ACUTE ON CHRONIC SYSTOLIC HEART FAILURE |
| V12.54 | cardiovascular | PERSONAL HISTORY OF TRANSIENT ISCHEMIC ATTACK (TIA), AND CEREBRAL INFARCTION WITHOUT RESIDUAL DEFICITS |
| 396.3 | cardiovascular | MITRAL VALVE INSUFFICIENCY AND AORTIC VALVE INSUFFICIENCY |
| 428.22 | cardiovascular | CHRONIC SYSTOLIC HEART FAILURE |
| V53.32 | cardiovascular | FITTING AND ADJUSTMENT OF AUTOMATIC IMPLANTABLE CARDIAC DEFIBRILLATOR |
| 397 | cardiovascular | DISEASES OF TRICUSPID VALVE |
| 427.1 | cardiovascular | PAROXYSMAL VENTRICULAR TACHYCARDIA |
| 427.69 | cardiovascular | OTHER PREMATURE BEATS |
| I42.0 | cardiovascular | DILATED CARDIOMYOPATHY |
| I47.2 | cardiovascular | VENTRICULAR TACHYCARDIA |
| 426.11 | cardiovascular | FIRST DEGREE ATRIOVENTRICULAR BLOCK |
| I10 | cardiovascular | ESSENTIAL (PRIMARY) HYPERTENSION |
| 393 | cardiovascular | CHRONIC RHEUMATIC PERICARDITIS |
| Z86.718 | cardiovascular | PERSONAL HISTORY OF OTHER VENOUS THROMBOSIS AND EMBOLISM |
| Z86.74 | cardiovascular | PERSONAL HISTORY OF SUDDEN CARDIAC ARREST |
| 424.1 | cardiovascular | AORTIC VALVE DISORDERS |
| 403.91 | cardiovascular | HYPERTENSIVE CHRONIC KIDNEY DISEASE, UNSPECIFIED, WITH CHRONIC KIDNEY DISEASE STAGE V OR END STAGE RENAL DISEASE |
| I12.0 | cardiovascular | HYPERTENSIVE CHRONIC KIDNEY DISEASE WITH STAGE 5 CHRONIC KIDNEY DISEASE OR END STAGE RENAL DISEASE |
| 426.7 | cardiovascular | ANOMALOUS ATRIOVENTRICULAR EXCITATION |
| Z95.0 | cardiovascular | PRESENCE OF CARDIAC PACEMAKER |
| I47.1 | cardiovascular | SUPRAVENTRICULAR TACHYCARDIA |
| I49.5 | cardiovascular | SICK SINUS SYNDROME |
| 426.6 | cardiovascular | OTHER HEART BLOCK |
| 427.81 | cardiovascular | SINOATRIAL NODE DYSFUNCTION |
| 429.1 | cardiovascular | MYOCARDIAL DEGENERATION |
| 416.8 | cardiovascular | OTHER CHRONIC PULMONARY HEART DISEASES |
| I27.2 | cardiovascular | OTHER SECONDARY PULMONARY HYPERTENSION |
| Z86.79 | cardiovascular | PERSONAL HISTORY OF OTHER DISEASES OF THE CIRCULATORY SYSTEM |
| 424.2 | cardiovascular | TRICUSPID VALVE DISORDERS, SPECIFIED AS NONRHEUMATIC |
| I34.1 | cardiovascular | NONRHEUMATIC MITRAL (VALVE) PROLAPSE |
| 403.9 | cardiovascular | HYPERTENSIVE CHRONIC KIDNEY DISEASE, UNSPECIFIED, WITH CHRONIC KIDNEY DISEASE STAGE I THROUGH STAGE IV, OR UNSPECIFIED |
| I12.9 | cardiovascular | HYPERTENSIVE CHRONIC KIDNEY DISEASE WITH STAGE 1 THROUGH STAGE 4 CHRONIC KIDNEY DISEASE, OR UNSPECIFIED CHRONIC KIDNEY DISEASE |
| I50.20 | cardiovascular | UNSPECIFIED SYSTOLIC (CONGESTIVE) HEART FAILURE |
| I50.23 | cardiovascular | ACUTE ON CHRONIC SYSTOLIC (CONGESTIVE) HEART FAILURE |
| Z95.2 | cardiovascular | PRESENCE OF PROSTHETIC HEART VALVE |
| 412 | cardiovascular | OLD MYOCARDIAL INFARCTION |
| 414.8 | cardiovascular | OTHER SPECIFIED FORMS OF CHRONIC ISCHEMIC HEART DISEASE |
| 427.2 | cardiovascular | PAROXYSMAL TACHYCARDIA, UNSPECIFIED |
| I46.9 | cardiovascular | CARDIAC ARREST, CAUSE UNSPECIFIED |
| I33.0 | cardiovascular | ACUTE AND SUBACUTE INFECTIVE ENDOCARDITIS |
| 416 | cardiovascular | PRIMARY PULMONARY HYPERTENSION |
| 427 | cardiovascular | PAROXYSMAL SUPRAVENTRICULAR TACHYCARDIA |
| 423.9 | cardiovascular | UNSPECIFIED DISEASE OF PERICARDIUM |
| I50.9 | cardiovascular | HEART FAILURE, UNSPECIFIED |
| I34.2 | cardiovascular | NONRHEUMATIC MITRAL (VALVE) STENOSIS |
| 420.9 | cardiovascular | ACUTE PERICARDITIS, UNSPECIFIED |
| I45.81 | cardiovascular | LONG QT SYNDROME |
| I49.49 | cardiovascular | OTHER PREMATURE DEPOLARIZATION |
| 401.1 | cardiovascular | BENIGN ESSENTIAL HYPERTENSION |
| I97.89 | cardiovascular | OTHER POSTPROCEDURAL COMPLICATIONS AND DISORDERS OF THE CIRCULATORY SYSTEM, NOT ELSEWHERE CLASSIFIED |
| Z95.810 | cardiovascular | PRESENCE OF AUTOMATIC (IMPLANTABLE) CARDIAC DEFIBRILLATOR |
| I50.21 | cardiovascular | ACUTE SYSTOLIC (CONGESTIVE) HEART FAILURE |
| I31.3 | cardiovascular | PERICARDIAL EFFUSION (NONINFLAMMATORY) |
| 427.9 | cardiovascular | CARDIAC DYSRHYTHMIA, UNSPECIFIED |
| 416.2 | cardiovascular | CHRONIC PULMONARY EMBOLISM |
| 414.01 | cardiovascular | CORONARY ATHEROSCLEROSIS OF NATIVE CORONARY ARTERY |
| 426 | cardiovascular | ATRIOVENTRICULAR BLOCK, COMPLETE |
| I44.2 | cardiovascular | ATRIOVENTRICULAR BLOCK, COMPLETE |
| 401 | cardiovascular | MALIGNANT ESSENTIAL HYPERTENSION |
| Z86.71 | cardiovascular | PERSONAL HISTORY OF VENOUS THROMBOSIS AND EMBOLISM |
| 413.9 | cardiovascular | OTHER AND UNSPECIFIED ANGINA PECTORIS |
| 428.32 | cardiovascular | CHRONIC DIASTOLIC HEART FAILURE |
| 428.33 | cardiovascular | ACUTE ON CHRONIC DIASTOLIC HEART FAILURE |
| 426.13 | cardiovascular | OTHER SECOND DEGREE ATRIOVENTRICULAR BLOCK |
| 427.32 | cardiovascular | ATRIAL FLUTTER |
| 428.21 | cardiovascular | ACUTE SYSTOLIC HEART FAILURE |
| 405.91 | cardiovascular | UNSPECIFIED RENOVASCULAR HYPERTENSION |
| 426.3 | cardiovascular | OTHER LEFT BUNDLE BRANCH BLOCK |
| 394.1 | cardiovascular | RHEUMATIC MITRAL INSUFFICIENCY |
| 396.1 | cardiovascular | MITRAL VALVE STENOSIS AND AORTIC VALVE INSUFFICIENCY |
| 398.91 | cardiovascular | RHEUMATIC HEART FAILURE (CONGESTIVE) |
| 394 | cardiovascular | MITRAL STENOSIS |
| I49.3 | cardiovascular | VENTRICULAR PREMATURE DEPOLARIZATION |
| I25.10 | cardiovascular | ATHEROSCLEROTIC HEART DISEASE OF NATIVE CORONARY ARTERY WITHOUT ANGINA PECTORIS |
| I07.1 | cardiovascular | RHEUMATIC TRICUSPID INSUFFICIENCY |
| I07.1 | cardiovascular | RHEUMATIC TRICUSPID INSUFFICIENCY |
| 413.1 | cardiovascular | PRINZMETAL ANGINA |
| 426.12 | cardiovascular | MOBITZ (TYPE) II ATRIOVENTRICULAR BLOCK |
| V12.53 | cardiovascular | PERSONAL HISTORY OF SUDDEN CARDIAC ARREST |
| 427.41 | cardiovascular | VENTRICULAR FIBRILLATION |
| 429.83 | cardiovascular | TAKOTSUBO SYNDROME |
| Z95.1 | cardiovascular | PRESENCE OF AORTOCORONARY BYPASS GRAFT |
| I45.5 | cardiovascular | OTHER SPECIFIED HEART BLOCK |
| 403 | cardiovascular | HYPERTENSIVE CHRONIC KIDNEY DISEASE, MALIGNANT, WITH CHRONIC KIDNEY DISEASE STAGE I THROUGH STAGE IV, OR UNSPECIFIED |
| I49.9 | cardiovascular | CARDIAC ARRHYTHMIA, UNSPECIFIED |
| 425.8 | cardiovascular | CARDIOMYOPATHY IN OTHER DISEASES CLASSIFIED ELSEWHERE |
| 416.9 | cardiovascular | CHRONIC PULMONARY HEART DISEASE, UNSPECIFIED |
| 420.91 | cardiovascular | ACUTE IDIOPATHIC PERICARDITIS |
| I48.91 | cardiovascular | UNSPECIFIED ATRIAL FIBRILLATION |
| I49.8 | cardiovascular | OTHER SPECIFIED CARDIAC ARRHYTHMIAS |
| 423.3 | cardiovascular | CARDIAC TAMPONADE |
| I44.0 | cardiovascular | ATRIOVENTRICULAR BLOCK, FIRST DEGREE |
| I44.1 | cardiovascular | ATRIOVENTRICULAR BLOCK, SECOND DEGREE |
| I49.1 | cardiovascular | ATRIAL PREMATURE DEPOLARIZATION |
| I16.1 | cardiovascular | HYPERTENSIVE EMERGENCY |
| 425.11 | cardiovascular | HYPERTROPHIC OBSTRUCTIVE CARDIOMYOPATHY |
| V53.31 | cardiovascular | FITTING AND ADJUSTMENT OF CARDIAC PACEMAKER |
| 426.89 | cardiovascular | OTHER SPECIFIED CONDUCTION DISORDERS |
| 429.5 | cardiovascular | RUPTURE OF CHORDAE TENDINEAE |
| I69.11 | cerebrovascular | COGNITIVE DEFICITS FOLLOWING NONTRAUMATIC INTRACEREBRAL HEMORRHAGE |
| I69.154 | cerebrovascular | HEMIPLEGIA AND HEMIPARESIS FOLLOWING NONTRAUMATIC INTRACEREBRAL HEMORRHAGE AFFECTING LEFT NON-DOMINANT SIDE |
| I63.9 | cerebrovascular | CEREBRAL INFARCTION, UNSPECIFIED |
| I61.9 | cerebrovascular | NONTRAUMATIC INTRACEREBRAL HEMORRHAGE, UNSPECIFIED |
| I60.7 | cerebrovascular | NONTRAUMATIC SUBARACHNOID HEMORRHAGE FROM UNSPECIFIED INTRACRANIAL ARTERY |
| I69.398 | cerebrovascular | OTHER SEQUELAE OF CEREBRAL INFARCTION |
| I67.848 | cerebrovascular | OTHER CEREBROVASCULAR VASOSPASM AND VASOCONSTRICTION |
| I63.8 | cerebrovascular | OTHER CEREBRAL INFARCTION |
| O99.413 | circulatory | DISEASES OF THE CIRCULATORY SYSTEM COMPLICATING PREGNANCY, THIRD TRIMESTER |
| I82.612 | circulatory | ACUTE EMBOLISM AND THROMBOSIS OF SUPERFICIAL VEINS OF LEFT UPPER EXTREMITY |
| O99.412 | circulatory | DISEASES OF THE CIRCULATORY SYSTEM COMPLICATING PREGNANCY, SECOND TRIMESTER |
| I95.1 | circulatory | ORTHOSTATIC HYPOTENSION |
| I96 | circulatory | GANGRENE, NOT ELSEWHERE CLASSIFIED |
| O99.411 | circulatory | DISEASES OF THE CIRCULATORY SYSTEM COMPLICATING PREGNANCY, FIRST TRIMESTER |
| I95.9 | circulatory | HYPOTENSION, UNSPECIFIED |
| I77.6 | circulatory | ARTERITIS, UNSPECIFIED |
| O99.43 | circulatory | DISEASES OF THE CIRCULATORY SYSTEM COMPLICATING THE PUERPERIUM |
| O99.42 | circulatory | DISEASES OF THE CIRCULATORY SYSTEM COMPLICATING CHILDBIRTH |
| I82.432 | circulatory | ACUTE EMBOLISM AND THROMBOSIS OF LEFT POPLITEAL VEIN |
| I82.B12 | circulatory | ACUTE EMBOLISM AND THROMBOSIS OF LEFT SUBCLAVIAN VEIN |
| I95.2 | circulatory | HYPOTENSION DUE TO DRUGS |
| V09.80 | drug-resistance | INFECTION WITH MICROORGANISMS WITHOUT MENTION OF RESISTANCE TO MULTIPLE DRUGS |
| Z16.19 | drug-resistance | RESISTANCE TO OTHER SPECIFIED BETA LACTAM ANTIBIOTICS |
| V09.91 | drug-resistance | INFECTION WITH DRUG-RESISTANT MICROORGANISMS, UNSPECIFIED, WITH MULTIPLE DRUG RESISTANCE |
| Z16.29 | drug-resistance | RESISTANCE TO OTHER SINGLE SPECIFIED ANTIBIOTIC |
| 8.8 | infectious disease | INTESTINAL INFECTION DUE TO OTHER ORGANISM, NOT ELSEWHERE CLASSIFIED |
| 99.53 | infectious disease | OTHER VENEREAL DISEASES DUE TO CHLAMYDIA TRACHOMATIS, LOWER GENITOURINARY SITES |
| 54.1 | infectious disease | GENITAL HERPES, UNSPECIFIED |
| 131.9 | infectious disease | TRICHOMONIASIS, UNSPECIFIED |
| V12.09 | infectious disease | PERSONAL HISTORY OF OTHER INFECTIOUS AND PARASITIC DISEASES |
| 112.1 | infectious disease | CANDIDIASIS OF VULVA AND VAGINA |
| 41.85 | infectious disease | OTHER SPECIFIED BACTERIAL INFECTIONS IN CONDITIONS CLASSIFIED ELSEWHERE AND OF UNSPECIFIED SITE, OTHER GRAM-NEGATIVE ORGANISMS |
| 41.4 | infectious disease | E. COLI INFECT NOS |
| 41.04 | infectious disease | STREPTOCOCCUS INFECTION IN CONDITIONS CLASSIFIED ELSEWHERE AND OF UNSPECIFIED SITE, STREPTOCOCCUS, GROUP D [ENTEROCOCCUS] |
| 79.99 | infectious disease | UNSPECIFIED VIRAL INFECTION |
| 41.49 | infectious disease | OTHER AND UNSPECIFIED ESCHERICHIA COLI [E. COLI] |
| 38.9 | infectious disease | UNSPECIFIED SEPTICEMIA |
| A60.00 | infectious disease | HERPESVIRAL INFECTION OF UROGENITAL SYSTEM, UNSPECIFIED |
| O98.313 | infectious disease | OTHER INFECTIONS WITH A PREDOMINANTLY SEXUAL MODE OF TRANSMISSION COMPLICATING PREGNANCY, THIRD TRIMESTER |
| 41.19 | infectious disease | STAPHYLOCOCCUS INFECTION IN CONDITIONS CLASSIFIED ELSEWHERE AND OF UNSPECIFIED SITE, OTHER STAPHYLOCOCCUS |
| 41.11 | infectious disease | METHICILLIN SUSCEPTIBLE STAPHYLOCOCCUS AUREUS IN CONDITIONS CLASSIFIED ELSEWHERE AND OF UNSPECIFIED SITE |
| 70.3 | infectious disease | VIRAL HEPATITIS B WITHOUT MENTION OF HEPATIC COMA, ACUTE OR UNSPECIFIED, WITHOUT MENTION OF HEPATITIS DELTA |
| 38.3 | infectious disease | SEPTICEMIA DUE TO ANAEROBES |
| 112.9 | infectious disease | CANDIDIASIS OF UNSPECIFIED SITE |
| 8.45 | infectious disease | INTESTINAL INFECTION DUE TO CLOSTRIDIUM DIFFICILE |
| 38.42 | infectious disease | SEPTICEMIA DUE TO ESCHERICHIA COLI [E. COLI] |
| 54.9 | infectious disease | HERPES SIMPLEX WITHOUT MENTION OF COMPLICATION |
| 41.9 | infectious disease | BACTERIAL INFECTION, UNSPECIFIED, IN CONDITIONS CLASSIFIED ELSEWHERE AND OF UNSPECIFIED SITE |
| 70.32 | infectious disease | CHRONIC VIRAL HEPATITIS B WITHOUT MENTION OF HEPATIC COMA WITHOUT MENTION OF HEPATITIS DELTA |
| 9.1 | infectious disease | COLITIS, ENTERITIS, AND GASTROENTERITIS OF PRESUMED INFECTIOUS ORIGIN |
| 70.7 | infectious disease | UNSPECIFIED VIRAL HEPATITIS C WITHOUT HEPATIC COMA |
| 70.54 | infectious disease | CHRONIC HEPATITIS C WITHOUT MENTION OF HEPATIC COMA |
| 79.4 | infectious disease | HUMAN PAPILLOMAVIRUS IN CONDITIONS CLASSIFIED ELSEWHERE AND OF UNSPECIFIED SITE |
| 41.12 | infectious disease | METHICILLIN RESISTANT STAPHYLOCOCCUS AUREUS IN CONDITIONS CLASSIFIED ELSEWHERE AND OF UNSPECIFIED SITE |
| 77.99 | infectious disease | UNSPECIFIED DISEASES OF CONJUNCTIVA DUE TO VIRUSES |
| 79.98 | infectious disease | UNSPECIFIED CHLAMYDIAL INFECTION |
| J18.9 | infectious disease | PNEUMONIA, UNSPECIFIED ORGANISM |
| 41.3 | infectious disease | FRIEDLDNDERS BACILLUS INFECTION IN CONDITIONS CLASSIFIED ELSEWHERE AND OF UNSPECIFIED SITE |
| 54.12 | infectious disease | HERPETIC ULCERATION OF VULVA |
| A41.9 | infectious disease | SEPSIS, UNSPECIFIED ORGANISM |
| J12.3 | infectious disease | HUMAN METAPNEUMOVIRUS PNEUMONIA |
| B00.1 | infectious disease | HERPESVIRAL VESICULAR DERMATITIS |
| B49 | infectious disease | UNSPECIFIED MYCOSIS |
| B37.0 | infectious disease | CANDIDAL STOMATITIS |
| B96.20 | infectious disease | UNSPECIFIED ESCHERICHIA COLI [E. COLI] AS THE CAUSE OF DISEASES CLASSIFIED ELSEWHERE |
| O98.813 | infectious disease | OTHER MATERNAL INFECTIOUS AND PARASITIC DISEASES COMPLICATING PREGNANCY, THIRD TRIMESTER |
| 52.9 | infectious disease | VARICELLA WITHOUT MENTION OF COMPLICATION |
| 27 | infectious disease | LISTERIOSIS |
| 41.02 | infectious disease | STREPTOCOCCUS INFECTION IN CONDITIONS CLASSIFIED ELSEWHERE AND OF UNSPECIFIED SITE, STREPTOCOCCUS, GROUP B |
| 41.6 | infectious disease | PROTEUS (MIRABILIS) (MORGANII) INFECTION IN CONDITIONS CLASSIFIED ELSEWHERE AND OF UNSPECIFIED SITE |
| 79.89 | infectious disease | OTHER SPECIFIED VIRAL INFECTION |
| B33.8 | infectious disease | OTHER SPECIFIED VIRAL DISEASES |
| O98.513 | infectious disease | OTHER VIRAL DISEASES COMPLICATING PREGNANCY, THIRD TRIMESTER |
| 98.19 | infectious disease | OTHER GONOCOCCAL INFECTION (ACUTE) OF UPPER GENITOURINARY TRACT |
| 41.7 | infectious disease | PSEUDOMONAS INFECTION IN CONDITIONS CLASSIFIED ELSEWHERE AND OF UNSPECIFIED SITE |
| 98 | infectious disease | GONOCOCCAL INFECTION (ACUTE) OF LOWER GENITOURINARY TRACT |
| 78.11 | infectious disease | CONDYLOMA ACUMINATUM |
| V12.01 | infectious disease | PERSONAL HISTORY OF TUBERCULOSIS |
| V12.04 | infectious disease | PERSONAL HISTORY OF METHICILLIN RESISTANT STAPHYLOCOCCUS AUREUS |
| B95.61 | infectious disease | METHICILLIN SUSCEPTIBLE STAPHYLOCOCCUS AUREUS INFECTION AS THE CAUSE OF DISEASES CLASSIFIED ELSEWHERE |
| A41.02 | infectious disease | SEPSIS DUE TO METHICILLIN RESISTANT STAPHYLOCOCCUS AUREUS |
| B00.9 | infectious disease | HERPESVIRAL INFECTION, UNSPECIFIED |
| 70.1 | infectious disease | VIRAL HEPATITIS A WITHOUT MENTION OF HEPATIC COMA |
| O98.511 | infectious disease | OTHER VIRAL DISEASES COMPLICATING PREGNANCY, FIRST TRIMESTER |
| 99.54 | infectious disease | OTHER VENEREAL DISEASES DUE TO CHLAMYDIA TRACHOMATIS, OTHER GENITOURINARY SITES |
| 41.1 | infectious disease | STAPHYLOCOCCUS INFECTION IN CONDITIONS CLASSIFIED ELSEWHERE AND OF UNSPECIFIED SITE, STAPHYLOCOCCUS, UNSPECIFIED |
| O98.812 | infectious disease | OTHER MATERNAL INFECTIOUS AND PARASITIC DISEASES COMPLICATING PREGNANCY, SECOND TRIMESTER |
| B95.62 | infectious disease | METHICILLIN RESISTANT STAPHYLOCOCCUS AUREUS INFECTION AS THE CAUSE OF DISEASES CLASSIFIED ELSEWHERE |
| O98.413 | infectious disease | VIRAL HEPATITIS COMPLICATING PREGNANCY, THIRD TRIMESTER |
| B00.3 | infectious disease | HERPESVIRAL MENINGITIS |
| O98.512 | infectious disease | OTHER VIRAL DISEASES COMPLICATING PREGNANCY, SECOND TRIMESTER |
| 131.01 | infectious disease | TRICHOMONAL VULVOVAGINITIS |
| 34 | infectious disease | STREPTOCOCCAL SORE THROAT |
| B96.1 | infectious disease | KLEBSIELLA PNEUMONIAE [K. PNEUMONIAE] AS THE CAUSE OF DISEASES CLASSIFIED ELSEWHERE |
| O98.32 | infectious disease | OTHER INFECTIONS WITH A PREDOMINANTLY SEXUAL MODE OF TRANSMISSION COMPLICATING CHILDBIRTH |
| 38.43 | infectious disease | SEPTICEMIA DUE TO PSEUDOMONAS |
| 38.49 | infectious disease | OTHER SEPTICEMIA DUE TO GRAM-NEGATIVE ORGANISMS |
| 112.2 | infectious disease | CANDIDIASIS OF OTHER UROGENITAL SITES |
| 84 | infectious disease | FALCIPARUM MALARIA [MALIGNANT TERTIAN] |
| 136.8 | infectious disease | OTHER SPECIFIED INFECTIOUS AND PARASITIC DISEASES |
| 49 | infectious disease | LYMPHOCYTIC CHORIOMENINGITIS |
| J10.00 | infectious disease | INFLUENZA DUE TO OTHER IDENTIFIED INFLUENZA VIRUS WITH UNSPECIFIED TYPE OF PNEUMONIA |
| 99.5 | infectious disease | OTHER VENEREAL DISEASES DUE TO CHLAMYDIA TRACHOMATIS, UNSPECIFIED SITE |
| B95.1 | infectious disease | STREPTOCOCCUS, GROUP B, AS THE CAUSE OF DISEASES CLASSIFIED ELSEWHERE |
| 31.9 | infectious disease | UNSPECIFIED DISEASES DUE TO MYCOBACTERIA |
| B96.89 | infectious disease | OTHER SPECIFIED BACTERIAL AGENTS AS THE CAUSE OF DISEASES CLASSIFIED ELSEWHERE |
| B96.81 | infectious disease | HELICOBACTER PYLORI [H. PYLORI] AS THE CAUSE OF DISEASES CLASSIFIED ELSEWHERE |
| A04.8 | infectious disease | OTHER SPECIFIED BACTERIAL INTESTINAL INFECTIONS |
| B19.20 | infectious disease | UNSPECIFIED VIRAL HEPATITIS C WITHOUT HEPATIC COMA |
| O98.42 | infectious disease | VIRAL HEPATITIS COMPLICATING CHILDBIRTH |
| 31 | infectious disease | PULMONARY DISEASES DUE TO OTHER MYCOBACTERIA |
| O98.312 | infectious disease | OTHER INFECTIONS WITH A PREDOMINANTLY SEXUAL MODE OF TRANSMISSION COMPLICATING PREGNANCY, SECOND TRIMESTER |
| 112 | infectious disease | CANDIDIASIS OF MOUTH |
| J11.1 | infectious disease | INFLUENZA DUE TO UNIDENTIFIED INFLUENZA VIRUS WITH OTHER RESPIRATORY MANIFESTATIONS |
| 112.3 | infectious disease | CANDIDIASIS OF SKIN AND NAILS |
| A59.9 | infectious disease | TRICHOMONIASIS, UNSPECIFIED |
| B37.3 | infectious disease | CANDIDIASIS OF VULVA AND VAGINA |
| 47.9 | infectious disease | UNSPECIFIED VIRAL MENINGITIS |
| 79.83 | infectious disease | PARVOVIRUS B19 |
| J11.00 | infectious disease | INFLUENZA DUE TO UNIDENTIFIED INFLUENZA VIRUS WITH UNSPECIFIED TYPE OF PNEUMONIA |
| B34.9 | infectious disease | VIRAL INFECTION, UNSPECIFIED |
| 78.5 | infectious disease | CYTOMEGALOVIRAL DISEASE |
| A40.9 | infectious disease | STREPTOCOCCAL SEPSIS, UNSPECIFIED |
| V12.03 | infectious disease | PERSONAL HISTORY OF MALARIA |
| 123.1 | infectious disease | CYSTICERCOSIS |
| 98.86 | infectious disease | GONOCOCCAL PERITONITIS |
| 9 | infectious disease | INFECTIOUS COLITIS, ENTERITIS, AND GASTROENTERITIS |
| 131.8 | infectious disease | TRICHOMONIASIS OF OTHER SPECIFIED SITES |
| B34.8 | infectious disease | OTHER VIRAL INFECTIONS OF UNSPECIFIED SITE |
| A56.8 | infectious disease | SEXUALLY TRANSMITTED CHLAMYDIAL INFECTION OF OTHER SITES |
| V12.00 | infectious disease | PERSONAL HISTORY OF UNSPECIFIED INFECTIOUS AND PARASITIC DISEASE |
| 54.19 | infectious disease | OTHER GENITAL HERPES |
| O98.412 | infectious disease | VIRAL HEPATITIS COMPLICATING PREGNANCY, SECOND TRIMESTER |
| A41.01 | infectious disease | SEPSIS DUE TO METHICILLIN SUSCEPTIBLE STAPHYLOCOCCUS AUREUS |
| 78 | infectious disease | MOLLUSCUM CONTAGIOSUM |
| 112.89 | infectious disease | OTHER CANDIDIASIS OF OTHER SPECIFIED SITES |
| 47.8 | infectious disease | OTHER SPECIFIED VIRAL MENINGITIS |
| O98.82 | infectious disease | OTHER MATERNAL INFECTIOUS AND PARASITIC DISEASES COMPLICATING CHILDBIRTH |
| O98.811 | infectious disease | OTHER MATERNAL INFECTIOUS AND PARASITIC DISEASES COMPLICATING PREGNANCY, FIRST TRIMESTER |
| A60.09 | infectious disease | HERPESVIRAL INFECTION OF OTHER UROGENITAL TRACT |
| A08.4 | infectious disease | VIRAL INTESTINAL INFECTION, UNSPECIFIED |
| 8.43 | infectious disease | INTESTINAL INFECTION DUE TO CAMPYLOBACTER |
| O98.52 | infectious disease | OTHER VIRAL DISEASES COMPLICATING CHILDBIRTH |
| A41.51 | infectious disease | SEPSIS DUE TO ESCHERICHIA COLI [E. COLI] |
| 54 | infectious disease | ECZEMA HERPETICUM |
| 54.43 | infectious disease | HERPES SIMPLEX DISCIFORM KERATITIS |
| A08.11 | infectious disease | ACUTE GASTROENTEROPATHY DUE TO NORWALK AGENT |
| J09.X2 | infectious disease | INFLUENZA DUE TO IDENTIFIED NOVEL INFLUENZA A VIRUS WITH OTHER RESPIRATORY MANIFESTATIONS |
| 84.6 | infectious disease | MALARIA, UNSPECIFIED |
| O98.83 | infectious disease | OTHER MATERNAL INFECTIOUS AND PARASITIC DISEASES COMPLICATING THE PUERPERIUM |
| A04.7 | infectious disease | ENTEROCOLITIS DUE TO CLOSTRIDIUM DIFFICILE |
| A63.0 | infectious disease | ANOGENITAL (VENEREAL) WARTS |
| B37.9 | infectious disease | CANDIDIASIS, UNSPECIFIED |
| 41.89 | infectious disease | OTHER SPECIFIED BACTERIAL INFECTIONS IN CONDITIONS CLASSIFIED ELSEWHERE AND OF UNSPECIFIED SITE, OTHER SPECIFIED BACTERIA |
| 54.11 | infectious disease | HERPETIC VULVOVAGINITIS |
| 110.8 | infectious disease | DERMATOPHYTOSIS OF OTHER SPECIFIED SITES |
| A64 | infectious disease | UNSPECIFIED SEXUALLY TRANSMITTED DISEASE |
| 110.5 | infectious disease | DERMATOPHYTOSIS OF THE BODY |
| A56.11 | infectious disease | CHLAMYDIAL FEMALE PELVIC INFLAMMATORY DISEASE |
| 41.01 | infectious disease | STREPTOCOCCUS INFECTION IN CONDITIONS CLASSIFIED ELSEWHERE AND OF UNSPECIFIED SITE, STREPTOCOCCUS, GROUP A |
| 41.09 | infectious disease | STREPTOCOCCUS INFECTION IN CONDITIONS CLASSIFIED ELSEWHERE AND OF UNSPECIFIED SITE, OTHER STREPTOCOCCUS |
| J18.8 | infectious disease | OTHER PNEUMONIA, UNSPECIFIED ORGANISM |
| 66.42 | infectious disease | WEST NILE FEVER WITH OTHER NEUROLOGIC MANIFESTATION |
| 41.86 | infectious disease | HELICOBACTER PYLORI [H. PYLORI] |
| 3 | infectious disease | SALMONELLA GASTROENTERITIS |
| B37.49 | infectious disease | OTHER UROGENITAL CANDIDIASIS |
| 54.72 | infectious disease | HERPES SIMPLEX MENINGITIS |
| 131 | infectious disease | UROGENITAL TRICHOMONIASIS, UNSPECIFIED |
| 41 | infectious disease | STREPTOCOCCUS INFECTION IN CONDITIONS CLASSIFIED ELSEWHERE AND OF UNSPECIFIED SITE, STREPTOCOCCUS, UNSPECIFIED |
| B95.2 | infectious disease | ENTEROCOCCUS AS THE CAUSE OF DISEASES CLASSIFIED ELSEWHERE |
| 41.84 | infectious disease | OTHER SPECIFIED BACTERIAL INFECTIONS IN CONDITIONS CLASSIFIED ELSEWHERE AND OF UNSPECIFIED SITE, OTHER ANAEROBES |
| 135 | infectious disease | SARCOIDOSIS |
| A63.8 | infectious disease | OTHER SPECIFIED PREDOMINANTLY SEXUALLY TRANSMITTED DISEASES |
| Z86.19 | infectious disease | PERSONAL HISTORY OF OTHER INFECTIOUS AND PARASITIC DISEASES |
| J10.1 | infectious disease | INFLUENZA DUE TO OTHER IDENTIFIED INFLUENZA VIRUS WITH OTHER RESPIRATORY MANIFESTATIONS |
| 9.2 | infectious disease | INFECTIOUS DIARRHEA |
| 111.9 | infectious disease | DERMATOMYCOSIS, UNSPECIFIED |
| 84.4 | infectious disease | OTHER MALARIA |
| 133 | infectious disease | SCABIES |
| B95.4 | infectious disease | OTHER STREPTOCOCCUS AS THE CAUSE OF DISEASES CLASSIFIED ELSEWHERE |
| 136.3 | infectious disease | PNEUMOCYSTOSIS |
| J12.9 | infectious disease | VIRAL PNEUMONIA, UNSPECIFIED |
| 99.59 | infectious disease | OTHER VENEREAL DISEASES DUE TO CHLAMYDIA TRACHOMATIS, OTHER SPECIFIED SITE |
| 92.9 | infectious disease | EARLY SYPHILIS, LATENT, UNSPECIFIED |
| B36.9 | infectious disease | SUPERFICIAL MYCOSIS, UNSPECIFIED |
| 131.09 | infectious disease | OTHER UROGENITAL TRICHOMONIASIS |
| A74.9 | infectious disease | CHLAMYDIAL INFECTION, UNSPECIFIED |
| A59.01 | infectious disease | TRICHOMONAL VULVOVAGINITIS |
| 40.82 | infectious disease | TOXIC SHOCK SYNDROME |
| 136.9 | infectious disease | UNSPECIFIED INFECTIOUS AND PARASITIC DISEASES |
| 97.9 | infectious disease | SYPHILIS, UNSPECIFIED |
| 96 | infectious disease | LATE SYPHILIS, LATENT |
| B59 | infectious disease | PNEUMOCYSTOSIS |
| 8.63 | infectious disease | ENTERITIS DUE TO NORWALK VIRUS |
| 136.1 | infectious disease | BEHCETS SYNDROME |
| 53.9 | infectious disease | HERPES ZOSTER WITHOUT MENTION OF COMPLICATION |
| 38.8 | infectious disease | OTHER SPECIFIED SEPTICEMIAS |
| J10.08 | infectious disease | INFLUENZA DUE TO OTHER IDENTIFIED INFLUENZA VIRUS WITH OTHER SPECIFIED PNEUMONIA |
| J09.X3 | infectious disease | INFLUENZA DUE TO IDENTIFIED NOVEL INFLUENZA A VIRUS WITH GASTROINTESTINAL MANIFESTATIONS |
| B02.9 | infectious disease | ZOSTER WITHOUT COMPLICATIONS |
| B18.1 | infectious disease | CHRONIC VIRAL HEPATITIS B WITHOUT DELTA-AGENT |
| B19.10 | infectious disease | UNSPECIFIED VIRAL HEPATITIS B WITHOUT HEPATIC COMA |
| B35.3 | infectious disease | TINEA PEDIS |
| 111 | infectious disease | PITYRIASIS VERSICOLOR |
| B37.89 | infectious disease | OTHER SITES OF CANDIDIASIS |
| 38 | infectious disease | STREPTOCOCCAL SEPTICEMIA |
| B96.5 | infectious disease | PSEUDOMONAS (AERUGINOSA) (MALLEI) (PSEUDOMALLEI) AS THE CAUSE OF DISEASES CLASSIFIED ELSEWHERE |
| B30.0 | infectious disease | KERATOCONJUNCTIVITIS DUE TO ADENOVIRUS |
| 117.3 | infectious disease | ASPERGILLOSIS |
| 8.49 | infectious disease | INTESTINAL INFECTION DUE TO OTHER ORGANISMS |
| B97.89 | infectious disease | OTHER VIRAL AGENTS AS THE CAUSE OF DISEASES CLASSIFIED ELSEWHERE |
| 99.55 | infectious disease | OTHER VENEREAL DISEASES DUE TO CHLAMYDIA TRACHOMATIS, UNSPECIFIED GENITOURINARY SITE |
| 112.84 | infectious disease | CANDIDAL ESOPHAGITIS |
| 117.9 | infectious disease | OTHER AND UNSPECIFIED MYCOSES |
| O41.1230 | maternal care | CHORIOAMNIONITIS, THIRD TRIMESTER, NOT APPLICABLE OR UNSPECIFIED |
| O36.5930 | maternal care | MATERNAL CARE FOR OTHER KNOWN OR SUSPECTED POOR FETAL GROWTH, THIRD TRIMESTER, NOT APPLICABLE OR UNSPECIFIED |
| O36.8130 | maternal care | DECREASED FETAL MOVEMENTS, THIRD TRIMESTER, NOT APPLICABLE OR UNSPECIFIED |
| O36.5932 | maternal care | MATERNAL CARE FOR OTHER KNOWN OR SUSPECTED POOR FETAL GROWTH, THIRD TRIMESTER, FETUS 2 |
| O36.5920 | maternal care | MATERNAL CARE FOR OTHER KNOWN OR SUSPECTED POOR FETAL GROWTH, SECOND TRIMESTER, NOT APPLICABLE OR UNSPECIFIED |
| O41.03X0 | maternal care | OLIGOHYDRAMNIOS, THIRD TRIMESTER, NOT APPLICABLE OR UNSPECIFIED |
| O34.33 | maternal care | MATERNAL CARE FOR CERVICAL INCOMPETENCE, THIRD TRIMESTER |
| O34.83 | maternal care | MATERNAL CARE FOR OTHER ABNORMALITIES OF PELVIC ORGANS, THIRD TRIMESTER |
| O32.1XX0 | maternal care | MATERNAL CARE FOR BREECH PRESENTATION, NOT APPLICABLE OR UNSPECIFIED |
| O35.1XX0 | maternal care | MATERNAL CARE FOR (SUSPECTED) CHROMOSOMAL ABNORMALITY IN FETUS, NOT APPLICABLE OR UNSPECIFIED |
| O34.32 | maternal care | MATERNAL CARE FOR CERVICAL INCOMPETENCE, SECOND TRIMESTER |
| O36.8120 | maternal care | DECREASED FETAL MOVEMENTS, SECOND TRIMESTER, NOT APPLICABLE OR UNSPECIFIED |
| O32.1XX2 | maternal care | MATERNAL CARE FOR BREECH PRESENTATION, FETUS 2 |
| O40.3XX0 | maternal care | POLYHYDRAMNIOS, THIRD TRIMESTER, NOT APPLICABLE OR UNSPECIFIED |
| O36.0930 | maternal care | MATERNAL CARE FOR OTHER RHESUS ISOIMMUNIZATION, THIRD TRIMESTER, NOT APPLICABLE OR UNSPECIFIED |
| O36.1930 | maternal care | MATERNAL CARE FOR OTHER ISOIMMUNIZATION, THIRD TRIMESTER, NOT APPLICABLE OR UNSPECIFIED |
| O34.82 | maternal care | MATERNAL CARE FOR OTHER ABNORMALITIES OF PELVIC ORGANS, SECOND TRIMESTER |
| O35.8XX0 | maternal care | MATERNAL CARE FOR OTHER (SUSPECTED) FETAL ABNORMALITY AND DAMAGE, NOT APPLICABLE OR UNSPECIFIED |
| O34.12 | maternal care | MATERNAL CARE FOR BENIGN TUMOR OF CORPUS UTERI, SECOND TRIMESTER |
| O34.42 | maternal care | MATERNAL CARE FOR OTHER ABNORMALITIES OF CERVIX, SECOND TRIMESTER |
| O36.63X0 | maternal care | MATERNAL CARE FOR EXCESSIVE FETAL GROWTH, THIRD TRIMESTER, NOT APPLICABLE OR UNSPECIFIED |
| O36.4XX0 | maternal care | MATERNAL CARE FOR INTRAUTERINE DEATH, NOT APPLICABLE OR UNSPECIFIED |
| O32.8XX0 | maternal care | MATERNAL CARE FOR OTHER MALPRESENTATION OF FETUS, NOT APPLICABLE OR UNSPECIFIED |
| O36.8990 | maternal care | MATERNAL CARE FOR OTHER SPECIFIED FETAL PROBLEMS, UNSPECIFIED TRIMESTER, NOT APPLICABLE OR UNSPECIFIED |
| O34.13 | maternal care | MATERNAL CARE FOR BENIGN TUMOR OF CORPUS UTERI, THIRD TRIMESTER |
| O41.8X30 | maternal care | OTHER SPECIFIED DISORDERS OF AMNIOTIC FLUID AND MEMBRANES, THIRD TRIMESTER, NOT APPLICABLE OR UNSPECIFIED |
| O32.1XX1 | maternal care | MATERNAL CARE FOR BREECH PRESENTATION, FETUS 1 |
| O35.0XX0 | maternal care | MATERNAL CARE FOR (SUSPECTED) CENTRAL NERVOUS SYSTEM MALFORMATION IN FETUS, NOT APPLICABLE OR UNSPECIFIED |
| O32.6XX0 | maternal care | MATERNAL CARE FOR COMPOUND PRESENTATION, NOT APPLICABLE OR UNSPECIFIED |
| O36.5931 | maternal care | MATERNAL CARE FOR OTHER KNOWN OR SUSPECTED POOR FETAL GROWTH, THIRD TRIMESTER, FETUS 1 |
| O34.93 | maternal care | MATERNAL CARE FOR ABNORMALITY OF PELVIC ORGAN, UNSPECIFIED, THIRD TRIMESTER |
| O34.593 | maternal care | MATERNAL CARE FOR OTHER ABNORMALITIES OF GRAVID UTERUS, THIRD TRIMESTER |
| O34.592 | maternal care | MATERNAL CARE FOR OTHER ABNORMALITIES OF GRAVID UTERUS, SECOND TRIMESTER |
| O34.03 | maternal care | MATERNAL CARE FOR UNSPECIFIED CONGENITAL MALFORMATION OF UTERUS, THIRD TRIMESTER |
| O34.30 | maternal care | MATERNAL CARE FOR CERVICAL INCOMPETENCE, UNSPECIFIED TRIMESTER |
| O36.8121 | maternal care | DECREASED FETAL MOVEMENTS, SECOND TRIMESTER, FETUS 1 |
| O41.1220 | maternal care | CHORIOAMNIONITIS, SECOND TRIMESTER, NOT APPLICABLE OR UNSPECIFIED |
| O36.5990 | maternal care | MATERNAL CARE FOR OTHER KNOWN OR SUSPECTED POOR FETAL GROWTH, UNSPECIFIED TRIMESTER, NOT APPLICABLE OR UNSPECIFIED |
| O32.2XX0 | maternal care | MATERNAL CARE FOR TRANSVERSE AND OBLIQUE LIE, NOT APPLICABLE OR UNSPECIFIED |
| O36.0130 | maternal care | MATERNAL CARE FOR ANTI-D [RH] ANTIBODIES, THIRD TRIMESTER, NOT APPLICABLE OR UNSPECIFIED |
| O36.5922 | maternal care | MATERNAL CARE FOR OTHER KNOWN OR SUSPECTED POOR FETAL GROWTH, SECOND TRIMESTER, FETUS 2 |
| O32.8XX1 | maternal care | MATERNAL CARE FOR OTHER MALPRESENTATION OF FETUS, FETUS 1 |
| O34.523 | maternal care | MATERNAL CARE FOR PROLAPSE OF GRAVID UTERUS, THIRD TRIMESTER |
| O36.8920 | maternal care | MATERNAL CARE FOR OTHER SPECIFIED FETAL PROBLEMS, SECOND TRIMESTER, NOT APPLICABLE OR UNSPECIFIED |
| O34.81 | maternal care | MATERNAL CARE FOR OTHER ABNORMALITIES OF PELVIC ORGANS, FIRST TRIMESTER |
| O36.5130 | maternal care | MATERNAL CARE FOR KNOWN OR SUSPECTED PLACENTAL INSUFFICIENCY, THIRD TRIMESTER, NOT APPLICABLE OR UNSPECIFIED |
| O36.5933 | maternal care | MATERNAL CARE FOR OTHER KNOWN OR SUSPECTED POOR FETAL GROWTH, THIRD TRIMESTER, FETUS 3 |
| O32.0XX0 | maternal care | MATERNAL CARE FOR UNSTABLE LIE, NOT APPLICABLE OR UNSPECIFIED |
| O41.8X21 | maternal care | OTHER SPECIFIED DISORDERS OF AMNIOTIC FLUID AND MEMBRANES, SECOND TRIMESTER, FETUS 1 |
| O41.03X2 | maternal care | OLIGOHYDRAMNIOS, THIRD TRIMESTER, FETUS 2 |
| O40.3XX1 | maternal care | POLYHYDRAMNIOS, THIRD TRIMESTER, FETUS 1 |
| O36.0920 | maternal care | MATERNAL CARE FOR OTHER RHESUS ISOIMMUNIZATION, SECOND TRIMESTER, NOT APPLICABLE OR UNSPECIFIED |
| O36.0120 | maternal care | MATERNAL CARE FOR ANTI-D [RH] ANTIBODIES, SECOND TRIMESTER, NOT APPLICABLE OR UNSPECIFIED |
| O36.8930 | maternal care | MATERNAL CARE FOR OTHER SPECIFIED FETAL PROBLEMS, THIRD TRIMESTER, NOT APPLICABLE OR UNSPECIFIED |
| O34.31 | maternal care | MATERNAL CARE FOR CERVICAL INCOMPETENCE, FIRST TRIMESTER |
| V14.0 | medication allergy | PERSONAL HISTORY OF ALLERGY TO PENICILLIN |
| V14.8 | medication allergy | PERSONAL HISTORY OF ALLERGY TO OTHER SPECIFIED MEDICINAL AGENTS |
| V14.1 | medication allergy | PERSONAL HISTORY OF ALLERGY TO OTHER ANTIBIOTIC AGENT |
| V14.6 | medication allergy | PERSONAL HISTORY OF ALLERGY TO ANALGESIC AGENT |
| V14.2 | medication allergy | PERSONAL HISTORY OF ALLERGY TO SULFONAMIDES |
| V14.3 | medication allergy | PERSONAL HISTORY OF ALLERGY TO OTHER ANTI-INFECTIVE AGENT |
| V14.5 | medication allergy | PERSONAL HISTORY OF ALLERGY TO NARCOTIC AGENT |
| V14.7 | medication allergy | PERSONAL HISTORY OF ALLERGY TO SERUM OR VACCINE |
| Z88.5 | medication allergy | ALLERGY STATUS TO NARCOTIC AGENT STATUS |
| Z88.0 | medication allergy | ALLERGY STATUS TO PENICILLIN |
| V14.4 | medication allergy | PERSONAL HISTORY OF ALLERGY TO ANESTHETIC AGENT |
| Z88.2 | medication allergy | ALLERGY STATUS TO SULFONAMIDES STATUS |
| Z88.8 | medication allergy | ALLERGY STATUS TO OTHER DRUGS, MEDICAMENTS AND BIOLOGICAL SUBSTANCES STATUS |
| Z88.1 | medication allergy | ALLERGY STATUS TO OTHER ANTIBIOTIC AGENTS STATUS |
| Z88.6 | medication allergy | ALLERGY STATUS TO ANALGESIC AGENT STATUS |
| 278 | obesity | OBESITY, UNSPECIFIED |
| V85.32 | obesity | BODY MASS INDEX 32.0-32.9, ADULT |
| V85.39 | obesity | BODY MASS INDEX 39.0-39.9, ADULT |
| 278.01 | obesity | MORBID OBESITY |
| V85.44 | obesity | BODY MASS INDEX 60.0-69.9, ADULT |
| V85.41 | obesity | BODY MASS INDEX 40.0-44.9, ADULT |
| V85.45 | obesity | BODY MASS INDEX 70 AND OVER, ADULT |
| V85.42 | obesity | BODY MASS INDEX 45.0-49.9, ADULT |
| O99.211 | obesity | OBESITY COMPLICATING PREGNANCY, FIRST TRIMESTER |
| Z68.42 | obesity | BODY MASS INDEX (BMI) 45.0-49.9, ADULT |
| V85.37 | obesity | BODY MASS INDEX 37.0-37.9, ADULT |
| Z68.41 | obesity | BODY MASS INDEX (BMI) 40.0-44.9, ADULT |
| O99.214 | obesity | OBESITY COMPLICATING CHILDBIRTH |
| V85.35 | obesity | BODY MASS INDEX 35.0-35.9, ADULT |
| V85.34 | obesity | BODY MASS INDEX 34.0-34.9, ADULT |
| V85.4 | obesity | BMI 40 AND OVER,ADULT |
| O99.213 | obesity | OBESITY COMPLICATING PREGNANCY, THIRD TRIMESTER |
| Z68.43 | obesity | BODY MASS INDEX (BMI) 50-59.9 , ADULT |
| Z68.38 | obesity | BODY MASS INDEX (BMI) 38.0-38.9, ADULT |
| O99.212 | obesity | OBESITY COMPLICATING PREGNANCY, SECOND TRIMESTER |
| V85.43 | obesity | BODY MASS INDEX 50.0-59.9, ADULT |
| Z68.34 | obesity | BODY MASS INDEX (BMI) 34.0-34.9, ADULT |
| V85.38 | obesity | BODY MASS INDEX 38.0-38.9, ADULT |
| V85.36 | obesity | BODY MASS INDEX 36.0-36.9, ADULT |
| Z68.37 | obesity | BODY MASS INDEX (BMI) 37.0-37.9, ADULT |
| Z68.45 | obesity | BODY MASS INDEX (BMI) 70 OR GREATER, ADULT |
| V85.30 | obesity | BODY MASS INDEX 30.0-30.9, ADULT |
| Z68.36 | obesity | BODY MASS INDEX (BMI) 36.0-36.9, ADULT |
| Z68.35 | obesity | BODY MASS INDEX (BMI) 35.0-35.9, ADULT |
| Z68.39 | obesity | BODY MASS INDEX (BMI) 39.0-39.9, ADULT |
| V85.33 | obesity | BODY MASS INDEX 33.0-33.9, ADULT |
| V85.31 | obesity | BODY MASS INDEX 31.0-31.9, ADULT |
| Z68.33 | obesity | BODY MASS INDEX (BMI) 33.0-33.9, ADULT |
| Z68.44 | obesity | BODY MASS INDEX (BMI) 60.0-69.9, ADULT |
| Z68.32 | obesity | BODY MASS INDEX (BMI) 32.0-32.9, ADULT |
| O99.215 | obesity | OBESITY COMPLICATING THE PUERPERIUM |
| Z68.31 | obesity | BODY MASS INDEX (BMI) 31.0-31.9, ADULT |
| 278.02 | obesity | OVERWEIGHT |
| V23.49 | obstetric history | PREGNANCY WITH OTHER POOR OBSTETRIC HISTORY |
| V23.41 | obstetric history | PREGNANCY WITH HISTORY OF PRE-TERM LABOR |
| Z87.51 | obstetric history | PERSONAL HISTORY OF PRE-TERM LABOR |
| V13.21 | obstetric history | PERSONAL HISTORY OF PRE-TERM LABOR |
| O34.21 | obstetric history | MATERNAL CARE FOR SCAR FROM PREVIOUS CESAREAN DELIVERY |
| O34.219 | obstetric history | PREVIOUS CESAREAN DELIVERY, ANTEPARTUM CONDITION OR COMPLICATION |
| O34.211 | obstetric history | MATERNAL CARE FOR LOW TRANSVERSE SCAR FROM PREVIOUS CESAREAN DELIVERY |
| V13.29 | obstetric history | PERSONAL HISTORY OF OTHER GENITAL SYSTEM AND OBSTETRIC DISORDERS |
| Z87.59 | obstetric history | PERSONAL HISTORY OF OTHER COMPLICATIONS OF PREGNANCY, CHILDBIRTH AND THE PUERPERIUM |
| Z98.891 | obstetric history | HISTORY OF CESAREAN SECTION |
| V45.73 | organ | ACQUIRED ABSENCE OF KIDNEY |
| V45.72 | organ | ACQUIRED ABSENCE OF INTESTINE (LARGE) (SMALL) |
| V45.77 | organ | ACQUIRED ABSENCE OF ORGAN, GENITAL ORGANS |
| V42.5 | organ | CORNEA REPLACED BY TRANSPLANT |
| Y83.6 | organ | REMOVAL OF OTHER ORGAN (PARTIAL) (TOTAL) AS THE CAUSE OF ABNORMAL REACTION OF THE PATIENT, OR OF LATER COMPLICATION, WITHOUT MENTION OF MISADVENTURE AT THE TIME OF THE PROCEDURE |
| E878.0 | organ | SURGICAL OPERATION WITH TRANSPLANT OF WHOLE ORGAN CAUSING ABNORMAL PATIENT REACTION, OR LATER COMPLICATION, WITHOUT MENTION OF MISADVENTURE AT TIME OF OPERATION |
| 996.81 | organ | COMPLICATIONS OF TRANSPLANTED KIDNEY |
| 996.81 | organ | COMPLICATIONS OF TRANSPLANTED KIDNEY |
| V42.0 | organ | KIDNEY REPLACED BY TRANSPLANT |
| Z90.49 | organ | ACQUIRED ABSENCE OF OTHER SPECIFIED PARTS OF DIGESTIVE TRACT |
| V42.81 | organ | BONE MARROW REPLACED BY TRANSPLANT |
| Y83.0 | organ | SURGICAL OPERATION WITH TRANSPLANT OF WHOLE ORGAN AS THE CAUSE OF ABNORMAL REACTION OF THE PATIENT, OR OF LATER COMPLICATION, WITHOUT MENTION OF MISADVENTURE AT THE TIME OF THE PROCEDURE |
| Y83.0 | organ | SURGICAL OPERATION WITH TRANSPLANT OF WHOLE ORGAN AS THE CAUSE OF ABNORMAL REACTION OF THE PATIENT, OR OF LATER COMPLICATION, WITHOUT MENTION OF MISADVENTURE AT THE TIME OF THE PROCEDURE |
| T86.12 | organ | KIDNEY TRANSPLANT FAILURE |
| T86.13 | organ | KIDNEY TRANSPLANT INFECTION |
| V42.83 | organ | PANCREAS REPLACED BY TRANSPLANT |
| V42.7 | organ | LIVER REPLACED BY TRANSPLANT |
| V42.2 | organ | HEART VALVE REPLACED BY TRANSPLANT |
| Z90.81 | organ | ACQUIRED ABSENCE OF SPLEEN |
| Z90.411 | organ | ACQUIRED PARTIAL ABSENCE OF PANCREAS |
| Z90.410 | organ | ACQUIRED TOTAL ABSENCE OF PANCREAS |
| V42.1 | organ | HEART REPLACED BY TRANSPLANT |
| Z94.1 | organ | HEART TRANSPLANT STATUS |
| 996.82 | organ | COMPLICATIONS OF TRANSPLANTED LIVER |
| V45.87 | organ | TRANSPLANTED ORGAN REMOVAL STATUS |
| Z94.84 | organ | STEM CELLS TRANSPLANT STATUS |
| 83.75 | organ | TENDON TRANSFER OR TRANSPLANTATION |
| 51.23 | procedure | LAPAROSCOPIC CHOLECYSTECTOMY |
| 17.33 | procedure | LAPR RIGHT HEMICOLECTOMY |
| 17.36 | procedure | LAP SIGMOIDECTOMY |
| 22.42 | procedure | FRONTAL SINUSECTOMY |
| 22.63 | procedure | ETHMOIDECTOMY |
| 22.64 | procedure | SPHENOIDECTOMY |
| 26.31 | procedure | PARTIAL SIALOADENECTOMY |
| 26.32 | procedure | COMPLETE SIALOADENECTOMY |
| 28.2 | procedure | TONSILLECTOMY WITHOUT ADENOIDECTOMY |
| 28.7 | procedure | CONTROL OF HEMORRHAGE AFTER TONSILLECTOMY AND ADENOIDECTOMY |
| 41.5 | procedure | TOTAL SPLENECTOMY |
| 43.82 | procedure | LAP VERTICAL GASTRECTOMY |
| 43.89 | procedure | OTHER PARTIAL GASTRECTOMY |
| 45.42 | procedure | ENDOSCOPIC POLYPECTOMY OF LARGE INTESTINE |
| 47.01 | procedure | LAPAROSCOPIC APPENDECTOMY |
| 47.09 | procedure | OTHER APPENDECTOMY |
| 47.19 | procedure | OTHER INCIDENTAL APPENDECTOMY |
| 48.36 | procedure | [ENDOSCOPIC] POLYPECTOMY OF RECTUM |
| 50.3 | procedure | LOBECTOMY OF LIVER |
| 51.22 | procedure | CHOLECYSTECTOMY |
| 52.52 | procedure | DISTAL PANCREATECTOMY |
| 55.51 | procedure | NEPHROURETERECTOMY |
| 65.31 | procedure | LAPAROSCOPIC UNILATERAL OOPHORECTOMY |
| 65.39 | procedure | OTHER UNILATERAL OOPHORECTOMY |
| 65.41 | procedure | LAPAROSCOPIC UNILATERAL SALPINGO-OOPHORECTOMY |
| 65.49 | procedure | OTHER UNILATERAL SALPINGO-OOPHORECTOMY |
| 66.4 | procedure | TOTAL UNILATERAL SALPINGECTOMY |
| 66.62 | procedure | SALPINGECTOMY WITH REMOVAL OF TUBAL PREGNANCY |
| 66.69 | procedure | OTHER PARTIAL SALPINGECTOMY |
| 68.39 | procedure | OTHER AND UNSPECIFIED SUBTOTAL ABDOMINAL HYSTERECTOMY |
| 68.49 | procedure | LAPAROSCOPIC TOTAL ABDOMINAL HYSTERECTOMY |
| 77.85 | procedure | OTHER PARTIAL OSTECTOMY, FEMUR |
| 77.88 | procedure | OTHER PARTIAL OSTECTOMY, TARSALS AND METATARSALS |
| 80.71 | procedure | SYNOVECTOMY, SHOULDER |
| 83.44 | procedure | OTHER FASCIECTOMY |
| 83.45 | procedure | OTHER MYECTOMY |
| 85.23 | procedure | SUBTOTAL MASTECTOMY |
| 85.42 | procedure | BILATERAL SIMPLE MASTECTOMY |
| 85.43 | procedure | UNILATERAL EXTENDED SIMPLE MASTECTOMY |
| 29881 | procedure | ARTHROSCOPY, KNEE, SURGICAL; WITH MENISCECTOMY (MEDIAL OR LATERAL, INCLUDING ANY MENISCAL SHAVING) |
| 44970 | procedure | LAPAROSCOPY, SURGICAL, APPECTOMY |
| 59151 | procedure | LAPAROSCOPIC TREATMENT OF ECTOPIC PREGNANCY; WITH SALPINGECTOMY AND/OR OOPHORECTOMY |
| O99.513 | respiratory | DISEASES OF THE RESPIRATORY SYSTEM COMPLICATING PREGNANCY, THIRD TRIMESTER |
| O99.511 | respiratory | DISEASES OF THE RESPIRATORY SYSTEM COMPLICATING PREGNANCY, FIRST TRIMESTER |
| O99.512 | respiratory | DISEASES OF THE RESPIRATORY SYSTEM COMPLICATING PREGNANCY, SECOND TRIMESTER |
| J96.02 | respiratory | ACUTE RESPIRATORY FAILURE WITH HYPERCAPNIA |
| J06.9 | respiratory | ACUTE UPPER RESPIRATORY INFECTION, UNSPECIFIED |
| O99.52 | respiratory | DISEASES OF THE RESPIRATORY SYSTEM COMPLICATING CHILDBIRTH |
| J02.0 | respiratory | STREPTOCOCCAL PHARYNGITIS |
| J96.01 | respiratory | ACUTE RESPIRATORY FAILURE WITH HYPOXIA |
| J96.90 | respiratory | RESPIRATORY FAILURE, UNSPECIFIED, UNSPECIFIED WHETHER WITH HYPOXIA OR HYPERCAPNIA |
| O99.53 | respiratory | DISEASES OF THE RESPIRATORY SYSTEM COMPLICATING THE PUERPERIUM |
| J96.91 | respiratory | RESPIRATORY FAILURE, UNSPECIFIED WITH HYPOXIA |
| J01.90 | respiratory | ACUTE SINUSITIS, UNSPECIFIED |
| J20.9 | respiratory | ACUTE BRONCHITIS, UNSPECIFIED |
| 980 | toxic effect | TOXIC EFFECT OF ETHYL ALCOHOL |
| 989.89 | toxic effect | TOXIC EFFECT OF OTHER SUBSTANCE, CHIEFLY NONMEDICINAL AS TO SOURCE, NOT ELSEWHERE CLASSIFIED |
| 980.9 | toxic effect | TOXIC EFFECT OF UNSPECIFIED ALCOHOL |
| 989.9 | toxic effect | TOXIC EFFECT OF UNSPECIFIED SUBSTANCE, CHIEFLY NONMEDICINAL AS TO SOURCE |
| 987.8 | toxic effect | TOXIC EFFECT OF OTHER SPECIFIED GASES, FUMES, OR VAPORS |

**Table S4. Medications identified in COVID-19 clinical trials with corresponding National Clinical Trial codes**

| **Drug** | **Mechanism of Action** | **FDA Indication** | **Repurposed Indication** | **Clinical Trials** | **Number of Clinical Trials** |
| --- | --- | --- | --- | --- | --- |
| alteplase | tissue‐plasminogen activator | Treatment of acute ischemic stroke | acute respiratory distress syndrome (ARDS) | NCT04357730 | 1 |
| amoxicillin / clavulanate | inhibits bacterial protein synthesis | Broad spectrum antibiotic | antiviral and immunomodulating effects, community-acquired pneumonia | NCT04363060, NCT02735707 | 2 |
| aspirin | inhibition of cyclooxygenase | Pain reliever | Prophylaxis of COVID-19-induced coagulopathy | NCT04363840, NCT04410328, NCT04365309, NCT04343001, NCT04333407 | 5 |
| atovaquone | has broad-spectrum activity against Plasmodium spp., P. carinii | Prevention of/treatment of Pneumocystis jirovecii pneumonia | COVID-19 pneumonia | NCT04456153, NCT04339426 | 2 |
| azithromycin | inhibits bacterial protein synthesis | Broad spectrum antibiotic | antiviral and immunomodulating effects, community-acquired pneumonia | NCT04354428, NCT04339426, NCT04322396, NCT04369365, NCT04363060, NCT04339816, NCT04365582, NCT04381962, NCT04332107, NCT04441424, NCT04358068, NCT04361461, NCT04328272, NCT04335552, NCT04351919, NCT04338698, NCT04349410, NCT04341207, NCT04349592, NCT04476680, NCT04344379, NCT04365231, NCT04321278, NCT04322123, NCT04359095, NCT04390594, NCT04392128, NCT04329832, NCT04334382, NCT04354597, NCT04374903, NCT04345861, NCT04395768, NCT04347512, NCT04371406, NCT04348474, NCT04329572, NCT04334512, NCT04459702, NCT04374552, NCT04336332, NCT04363203, NCT04359316, NCT04355052, NCT04370782, NCT04405921, NCT04344444, NCT04328961, NCT04374019, NCT04458948, NCT04341727, NCT04383717, NCT04341870, NCT04332094 | 54 |
| budesonide | potent glucocorticoid activity and weak mineralocorticoid activity | Treatment of asthma, Crohn’s disease, ulcerative colitis | Inhibition of proinflammatory cytokine production | NCT04193878, NCT04422275, NCT04355637, NCT04361474, NCT04416399, NCT04331054, NCT04331470 | 7 |
| clindamycin | inhibits bacterial protein synthesis | Treatment of serious bacterial infection | COVID-19 pneumonia | NCT04349410 | 1 |
| co-trimoxazole (trimethoprim / sulfamethoxazole) | inhibits bacterial protein synthesis | respiratory infection antibiotic | Antimicrobial, immunomodulatory and anti-inflammatory | NCT04470531 | 1 |
| dexamethasone | Anti-inflammatory and immunosuppressive | Corticosterioid several medical usages | Anti-inflammatory | NCT04452565, NCT04360876, NCT04395105, NCT04344730, NCT04327401, NCT04325061, NCT04347980, NCT04476979 | 8 |
| diltiazem |  |  |  | NCT04372082 | 1 |
| dornase alfa | selectively cleaves DNA | management of cystic fibrosis | ARDS, hypoxia | NCT04402970, NCT04445285, NCT04355364, NCT04359654, NCT04432987, NCT04402944 | 6 |
| doxycycline | inhibits bacterial protein synthesis | Respiratory antibiotic | anti-inflammatory effect on cytokine expression and MMP activity | NCT04349410, NCT04371952 | 2 |
| enoxaparin | binds to and potentiates antithrombin | Prophylaxis of deep vein thrombosis | COVID-19 coagulopathy | NCT04373707, NCT04408235, NCT04366960, NCT04401293, NCT04367831, NCT04345848, NCT04377997, NCT04486508, NCT04359277, NCT04427098, NCT04400799, NCT04406389, NCT04394377, NCT04466670 | 14 |
| famotidine | H2 antagonist | Stomach ulcers, gastroesophageal reflux disease | Improve clinical outcomes of hospitalized COVID-19 patients | NCT04370262 | 1 |
| fluoxetine | Inhibits the serotonin transporter protein | Treatment of Major Depressive Disorder, obsessive compulsive disorder, bulimia nervosa, panic disorder | Reduce intubation and death after COVID-19 | NCT04377308 | 1 |
| folic acid | stimulates the production of red blood cells, white blood cells, and platelets | Treatment of folate deficiency megaloblastic anemia. | Reduction of viral replication | NCT04354428 | 1 |
| heparin | binds to the enzyme inhibitor antithrombin III | Prophylaxis and treatment of embolism | COVID-19 coagulopathy | NCT04373707, NCT04344756, NCT04345848, NCT04359277, NCT04366960, NCT04367831, NCT04377997, NCT04394377, NCT04400799, NCT04401293, NCT04406389, NCT04408235, NCT04409834, NCT04427098, NCT04466670, NCT04486508, NCT04487990 | 17 |
| hydrocortisone | Anti-inflammatory and immunosuppressive | Corticosteroid several medical usages | Anti-inflammatory, community-acquired pneumonia | NCT02735707, NCT04366115, NCT04348305, NCT04359511 | 4 |
| hydroxychloroquine | increases lysosomal pH in antigen-presenting cells | Malaria, rheumatoid arthritis, lupus Erythematosus | Prophylactic reduction of viral titer | NCT04371523, NCT04354428, NCT04322396, NCT04339816, NCT04365582, NCT04355026, NCT04303507, NCT04360759, NCT04351724, NCT04330586, NCT04334928, NCT04359615, NCT04441424, NCT04316377, NCT04358068, NCT04321616, NCT04361461, NCT04328272, NCT04333225, NCT04483960, NCT04352933, NCT04403100, NCT04466540, NCT04414241, NCT04325893, NCT04334148, NCT04328285, NCT04318444, NCT04335552, NCT04384458, NCT04349228, NCT04351919, NCT04346667, NCT04351191, NCT04346329, NCT04338698, NCT04349410, NCT04341207, NCT04345653, NCT04349592, NCT04341441, NCT04476680, NCT04344379, NCT04365231, NCT04321278, NCT04322123, NCT04410562, NCT04346147, NCT04359095, NCT04390594, NCT04392128, NCT04340544, NCT04315896, NCT04329832, NCT04334382, NCT04318015, NCT04333654, NCT04363827, NCT04331834, NCT04391127, NCT04354597, NCT04374903, NCT04382625, NCT04363450, NCT04343768, NCT04477083, NCT04381988, NCT04379492, NCT04377646, NCT04466280, NCT04350450, NCT04345861, NCT04395768, NCT04330495, NCT04437693, NCT04336748, NCT04443725, NCT04347512, NCT04371406, NCT04330144, NCT04400019, NCT04364815, NCT04371926, NCT04389359, NCT04354441, NCT04342156, NCT04397328, NCT04370015, NCT04347889, NCT04323631, NCT04372082, NCT04340349, NCT04328467, NCT04308668, NCT04358081, NCT04354870, NCT04363866, NCT04491994, NCT04344457, NCT04334967, NCT04408456, NCT04348474, NCT04329572, NCT04335084, NCT04334512, NCT04459702, NCT04353037, NCT04345692, NCT04438837, NCT04374552, NCT04387760, NCT04336332, NCT04363203, NCT04372017, NCT04359537, NCT04359316, NCT04261517, NCT04355052, NCT04394442, NCT04370782, NCT04369742, NCT04384380, NCT04352946, NCT04461353, NCT04385264, NCT04364022, NCT04374942, NCT04405921, NCT04344444, NCT04332991, NCT04329611, NCT04328961, NCT04351620, NCT04374019, NCT04421664, NCT04435808, NCT04458948, NCT04329923, NCT04353271, NCT04342169, NCT04341727, NCT04429867, NCT04342221, NCT04350671, NCT04383717, NCT04341493, NCT04303299, NCT04341870, NCT04332094, NCT04350684 | 151 |
| ibuprofen |  |  |  | NCT04382768, NCT04334629 | 2 |
| indomethacin | inhibition of cyclooxygenase | Rheumatoid arthritis, gouty arthritis, ankylosing spondylitis, tendonitis, osteoarthritis | Inhibition of viral replication | NCT04344457 | 1 |
| levofloxacin | inhibits bacterial protein synthesis | Broad spectrum antibiotic, including community-acquired pneumonia |  | NCT02735707 | 1 |
| mefloquine |  |  | COVID-19 pneumonia | NCT04347031 | 1 |
| methylprednisolone | Anti-inflammatory and immunosuppressive | Corticosteroid several medical usages | mitigates cytokine storm associated with COVID-19 | NCT04485429, NCT04263402, NCT04343729, NCT04438980, NCT03852537, NCT04244591, NCT04355247, NCT04341038, NCT04435717, NCT04345445 | 10 |
| oseltamivir | inhibitor of influenza virus neuraminidase | prophylaxis of influenza A and B | antiviral | NCT04371601, NCT04457609 | 2 |
| prednisone |  |  |  | NCT04344288, NCT04451174, NCT04359511 | 3 |
| propranolol hydrochloride |  |  |  | NCT04467086 | 1 |
| tacrolimus |  |  |  | NCT04341038 | 1 |
| tramadol |  |  |  | NCT04454307 | 1 |
| vitamin D | Increase intestinal absorption of calcium, magnesium, and phosphate | Vitamin D deficiency | Immune support | NCT04489628,  NCT04372017 |  |

**Table S5. Model Goodness of Fit Statistics for Final Models from the MWAS**

The goodness of fit statistics provided in the Supplemental Table 5 CSV file were completed with base r, using the glm() function for the following values: null deviance, deviance residuals, and Akaike information criterion (AIC). The caret package was used for the varImp() function to calculate variable importance of the medication in the model. Lastly, the AICcmodavg package was used to use the AICc() function to calculate corrected Akaike information criterion (AICc).
